# The Role of Gut Bacterial Metabolites in Brain Development, Aging and Disease

**DOI:** 10.3390/nu13030732

**Published:** 2021-02-25

**Authors:** Shirley Mei-Sin Tran, M. Hasan Mohajeri

**Affiliations:** Department of Medicine, Institute of Anatomy, University of Zurich, Winterthurerstrasse 190, 8057 Zürich, Switzerland; tran.shirleymeisin@gmail.com

**Keywords:** gut–brain–axis, gut microbiome, short-chain fatty acids, bacterial metabolites, SCFA

## Abstract

In the last decade, emerging evidence has reported correlations between the gut microbiome and human health and disease, including those affecting the brain. We performed a systematic assessment of the available literature focusing on gut bacterial metabolites and their associations with diseases of the central nervous system (CNS). The bacterial metabolites short-chain fatty acids (SCFAs) as well as non-SCFAs like amino acid metabolites (AAMs) and bacterial amyloids are described in particular. We found significantly altered SCFA levels in patients with autism spectrum disorder (ASD), affective disorders, multiple sclerosis (MS) and Parkinson’s disease (PD). Non-SCFAs yielded less significantly distinct changes in faecal levels of patients and healthy controls, with the majority of findings were derived from urinary and blood samples. Preclinical studies have implicated different bacterial metabolites with potentially beneficial as well as detrimental mechanisms in brain diseases. Examples include immunomodulation and changes in catecholamine production by histone deacetylase inhibition, anti-inflammatory effects through activity on the aryl hydrocarbon receptor and involvement in protein misfolding. Overall, our findings highlight the existence of altered bacterial metabolites in patients across various brain diseases, as well as potential neuroactive effects by which gut-derived SCFAs, p-cresol, indole derivatives and bacterial amyloids could impact disease development and progression. The findings summarized in this review could lead to further insights into the gut–brain–axis and thus into potential diagnostic, therapeutic or preventive strategies in brain diseases.

## 1. Introduction

We are exposed to bacterial organisms from the beginning of our existence to the end of it. Even before birth, bacteria have been detected in the meconium of newborns, thus discrediting the pre-existing idea of a sterile foetal stage [1]. Later on, the early postnatal exposure to either the mother’s vaginal flora or microbes from the environment, depending on delivery, impacts microbial colonization patterns, overall health and the neurodevelopment of the individual [2]. Although the microbial residents in our gastrointestinal tract (GIT) have already been known to impact the state of human health, the theory of a bidirectional gut–brain–axis (GBA) has taken the spotlight of global researchers mostly after the turn of the millennium.

**Figure 1:**
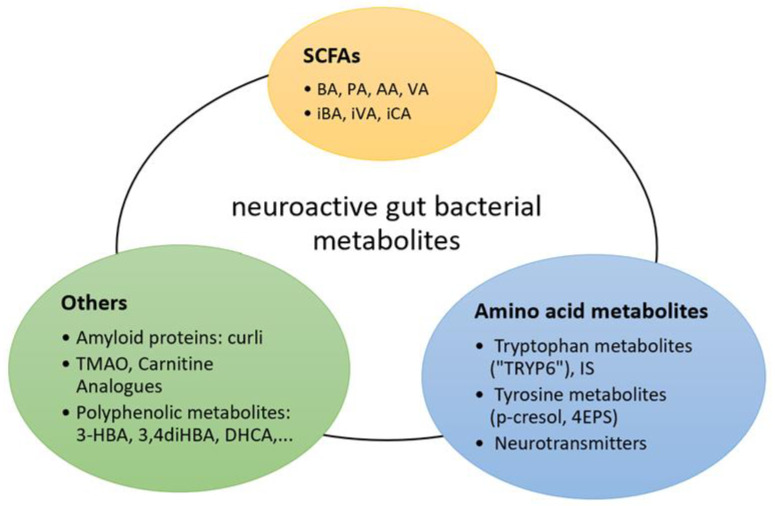
Non-exhaustive overview of neuroactive gut bacterial metabolites. SCFAs = short-chain fatty acids; BA = butyric acid; PA = propionic acid; AA = acetic acid; VA = valeric acid; iBA = isobutyric acid; iVA = isovaleric acid; iCA = isocaproic acid; TMAO = trimethylamine N-oxide; 3-HBA = 3-hydroxybenzoic acid; 3,4-diHBA = 3,4-dihydroxybenzoic acid; DHCA = dihydrocaffeic acid; IS = indoxyl sulphate; 4EPS = 4-ethylphenylsulfate.

Individuals are globally affected by increasing morbidity and mortality of psychiatric, neurodegenerative and neurodevelopmental disorders. The aetiology and pathophysiology of these brain diseases remain to this day to be fully elucidated and treatment options are largely of symptomatic nature. Therefore, researchers have unsurprisingly been looking at novel perspectives of disease, such as the GBA. Emerging findings on gut microbial influence on our nervous system were reported, involving bacterial-derived toxins, vitamins and neurotransmitters, yet the precise mechanisms, the “language of the GBA” [3], remain to be fully elucidated. Some newly examined neuroactive bacterial metabolites have nevertheless shown potential to play a role in this communication (Figure 1).

This systematic review intends to summarize the research on various families of neuroactive bacterial metabolites as probable key players in the GBA. The focus is their effects on disorders of the brain, ranging from neurodevelopmental stages in childhood to neurodegenerative diseases in advanced age. Although intriguing evidence has emerged about the GBA’s role in brain tumorigenesis via the modulation of the immune system, we refer the reader to a recent extensive study [4], as a detailed examination of this subject is beyond the scope of this review. Considering the magnitude of various influences from bacterial metabolites on the human organism, we will focus mostly on direct neuroactive effects on the brain. Most papers have largely emphasized taxonomic shifts in gut microbiota in specific diseases, or short-chain fatty acids (SCFAs) to date. One of our objectives is to provide a summary of findings between SCFAs and brain diseases, while in the second part of this review, reports of less explored non-SCFAs will take centre stage.

## 2. Materials and Methods 

This systematic review was conducted according to the Preferred Reporting Items for Systematic Reviews and Meta-Analyses (PRISMA) guidelines [5]. The main objective was to explore and summarize the available data on influences of gut bacterial metabolites on the brain, with a focus on neurodevelopmental, autoimmune-mediated neuroinflammatory, and neurodegenerative diseases. 

**Figure 2:**
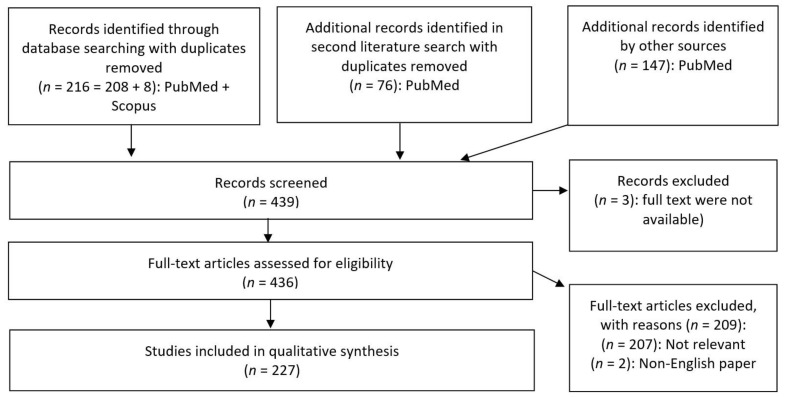
Methodical approach of our systematic review adhering to Preferred Reporting Items for Systematic Reviews and Meta-Analyses (PRISMA) criteria (PRISMA criteria [5]).

The first PubMed and SCOPUS databank searches were conducted on 20 November 2019. A second search was performed on the 8 July 2020 with the objective to include additional recently published data. The following search parameters and MeSH (Medical Subject Headings) terms “bacterial metabolites” combined with “brain development”, “brain aging”, “brain ageing”, “brain disorders”, “brain diseases”, “neurodegenerative”, “neuroprotective”, “gut brain axis” and “gut-brain-axis” delivered 216 hits after removing duplicates (Figure 2). The second search with the same search parameters delivered 76 new hits. One hundred and forty-seven additional records with relevant information were individually selected from the list of references of the initially identified papers. Our focus on gut bacterial metabolites warranted the exclusion of data on viruses, archaea, and fungi as well as data on bacteria not related to the gut microbiome. Original papers as well as reviews were included, while no restriction on publication year was applied. The inclusion criteria were the following:

- Published in a peer-reviewed article;
- Paper available in full-text PDF;
- Paper available in English;
- Paper discussing metabolites from bacteria found in gastrointestinal tracts of animals.

Most of the papers were dated from 2013 to 2019. Three papers lacking full texts, as well as two non-English publications, were excluded (Figure 2). Two hundred and seven papers were further excluded based on the lack of relevance to the topic. Finally, 227 studies were inspected for the qualitative synthesis. As to our knowledge, no other review to date has undertaken an analysis to this extent of links between several categories of gut bacterial metabolites and brain diseases. 

## 3. Short-Chain Fatty Acids

Short-chain fatty acids (SCFAs) are saturated fatty acids produced by the bacterial fermentation of dietary fibre [6]. The majority of SCFAs consist of acetic (AA), propionic (PA) and butyric acid (BA), which are mostly deprotonated in the intestine (acetate, propionate, butyrate) [7]. Some gut bacterial species capable of generating SCFAs are *Bacteroides*, *Bifidobacterium*, *Propionibacterium*, *Eubacterium*, *Lactobacillus*, *Clostridium*, *Roseburia* and *Prevotella.* Among them, *Roseburia*, *Eubacterium* and Lachnospiraceae (Firmicutes phylum, Clostridia class) are strong BA producers, while AA producers belong to the *Bifidobacteria spp.* [8]. Considering their production site, the initial point of contact with the human organism are colonocytes and other intestinal cells. This naturally leads to discussions of local impacts from SCFAs on overall gut health, predominantly in the context of diseases like irritable bowel syndrome (IBS) [9,10,11] and the inflammatory bowel diseases Morbus Crohn and Colitis Ulcerosa [12,13]. Local effects facilitated by SCFAs have previously been discussed in detail and will not be further elaborated in this review [12,14,15]. 

It is known that SCFAs are able to modulate gut permeability by upregulating tight junction proteins [16,17], which are also part of the blood–brain barrier (BBB). This conceivably raises the idea that barrier integrity of gut and brain could be similarly affected by SCFAs [18]. Indeed, studies in germ-free (GF) mice demonstrated that SCFAs are capable of modulating BBB permeability, which consequently impacts the extent to which beneficial or harmful molecules in circulation can reach brain tissue [19,20]. For example, physiological amounts of PA have been recently shown to protect the BBB from oxidative stress [21] and to decrease paracellular permeability [22]. Similarly, BA and BA-producing *Clostridium butyricum* can lower BBB permeability through enhancing tight-junction expression in mice [22]. In addition to directly affecting the BBB, SCFAs might actually reduce systemic inflammation by decreasing gut permeability, thereby decreasing circulating gut-derived bacterial components that trigger neuroinflammation by injuring the BBB or by affecting immune cells and cytokines in the brain [15]. SCFAs also act upon various gut–brain–pathways including immune, endocrine, vagal and direct humoral pathways (extensively reviewed by Dalile et al. [15]) and some effects in cellular systems, namely:

1. Histone deacetylase inhibition (HDACI) through BA, PA and AA, resulting in upregulated gene transcriptions in the context of epigenetic modulation [23,24]. As extensively reviewed by Stilling et al. [24] with a focus on BA, studies on this subject are mainly conducted in animal models and in supraphysiological concentrations, thus the validity of any conclusions drawn from the current evidence is promising, yet limited for human application as of now.
2. Agonistic effects on G-protein-coupled receptors (GPCRs), namely free fatty acid receptors FFAR2 (GPR43), FFAR3 (GPR41) and the niacin receptor 1 (NIACR1, also known as hydroxycarboxylic acid receptor 2 (HCAR2) or GPR109A) [15,25]. Whether these effects are relevant in humans is to be determined, since current findings on these GPCRs are mostly based on rodent or cell models. FFAR3 for example, was found in the CNS and sympathetic ganglia of rats, and in the peripheral nervous system of mice [15]. Moreover, results linking these GPCRs with microglia cell morphology and growth hormone secretion in pituitary cells [25,26] call for further research with a focus on SCFAs as potential bacterial mediators of brain function.
3. Modifications of cellular metabolism and activity in immune cells [27,28]. Similar to points 1 and 2, findings on these SCFA-mediated mechanisms are currently derived from animal and cell-based models. Nevertheless, studies have demonstrated striking results on BA promoting cell metabolism and differentiation in memory T cells [27,28], which underlines the importance of this mechanism.
4. Regulatory effects on transcription factors like peroxisome proliferator-activated receptor γ (PPAR-y) and hypoxia inducible factor-1 (HIF-1) [23,25,29] involved in inflammatory processes were also registered, though studies beyond rodents and cell models are currently lacking.

These studies have demonstrated SCFAs to be capable of regulating neuroinflammatory processes involving immune cell recruitment and cytokine secretion [29]. Microglia, immune cells residing in the CNS, were observed to be dysregulated in various psychiatric disorders like depression, schizophrenia, autism spectrum disorder (ASD) and obsessive–compulsive disorder [15] as well as in germ-free animals [28]. Interestingly, Toll-like receptors (TLRs) known to recognize bacterial compounds and to regulate inflammatory responses in our gastrointestinal tract (GIT) were found on various cell-types of the CNS, thus further supporting a link between gut and brain immune processes [30]. SCFAs also seem to directly impact neuronal function, as reported by studies showing PA and BA affecting intracellular potassium concentrations [31] and findings on influences on neurotransmitter metabolism [15]. Furthermore, beneficial effects on preserving memory function in experimental meningitis and protection from ouabain-induced hyperlocomotion were reported through a modulatory effect on the expression and activity of neurotrophic factors like brain-derived neurotrophic factor (BDNF), nerve growth factor (NGF) and glial cell line-derived neurotrophic factor (GDNF) in rats [32,33]. Interesting to note are the effects on synaptic plasticity by HDACI, since this process involves protein synthesis and therefore, gut-derived SCFAs might be potential epigenetic modulators of learning, memory formation and storage [34,35]. In light of these findings, altered SCFA production in the presence of gut microbiome disturbances, also known as dysbiosis, has been postulated as a potential risk for brain developmental and neurodegenerative diseases. Currently, CNS pathologies are often associated with changes in taxonomical gut microbiome and bacterial metabolites, as will be elaborated on in the following chapters. 

### 3.1. SCFA and Autism Spectrum Disorder

Autism spectrum disorder (ASD), a neurodevelopmental disorder characterized by behavioural abnormalities including repetitive behaviour, communication deficits and sensitivity to environmental changes, is often linked to gastrointestinal problems and alterations in the gut microbial community [36,37,38,39]. This was shown in a cohort of human infants, that distinct gut bacterial composition variations, at times called enterotypes, might correlate with cognitive performance [40]. Recent studies indeed reported the gut microbiome compositions of children with ASD to be significantly distinct from their neurotypical (NT) developing peers, and furthermore detected overall lower alpha diversity in ASD gut microbiomes [41]. 

Only a handful of human studies have measured faecal metabolites in ASD, with some of them reporting elevated [42] and others decreased total SCFA levels [39,43] in children with ASD (Table 1). Contrarily, Kang et al. [41] reported no significant differences in SCFAs levels between ASD and NT control group. Adams et al. [43] reported lower faecal levels across all SCFAs (AA, BA, PA, valeric acid (VA)) in children with ASD. Others observed lower levels of AA and BA, but no significant alterations in PA-levels [44]. Conversely, significantly increased faecal levels across all SCFAs (AA, BA, PA, VA, isobutyric and isovaleric acid) in one study [42], and significantly elevated AA and PA in another study were detected in ASD faecal samples [39,43]. In support of the findings on decreased BA in two of the studies mentioned, a metagenomic analysis on faecal samples resulted in a lower abundance of microbial genes involved in the production of BA [38], which also parallels a prior reported decrease in BA-producing *Faecalium prausnitzii* in autistic patients [41]. 

Supporting the idea of gut microbial influence on ASD development, Sharon et al. [45] demonstrated that faecal microbiome transplants (FMT) from human ASD donors were able to invoke ASD-like behavioural traits in mice. Moreover, El-Ansary et al. [46] reported neuronal DNA damage induced by PA oral administration in a hamster model. These suggested that PA could play a role in neurotoxicity by damaging mitochondrial DNA by ATP-depletion, thus leading to mitochondrial dysfunction and oxidative stress in neurons. This postulated pathway in autism has been underscored by earlier findings in rat pups exposed to PA, exhibiting various immune, mitochondrial and ASD-like behaviour changes similar to ASD in humans [46,47,48,49]. PA-induced ASD in rodents is a validated model for ASD research that has presented with abnormal neural cell organization and hippocampal histology, increased microglia activity, neurotoxic cytokine secretion, and typical ASD-like behaviour traits [50]. Moreover, perturbed microbiota with increased PA-producers and decreased BA producers correlated with the severity of disease burden in ASD [26,43], even if studies of PA faecal levels in children with ASD compared to healthy controls (HCs) have produced conflicting data [39,41].

Contrarily to PA, BA has shown overall beneficial effects in ASD. BA administration alleviated ASD-like behaviour and normalized changes in gene transcription related to inhibitory/excitatory balance in the frontal cortex of the T+tf/J strain of the black and tan brachyury (BTBR) mouse autism model [51]. Nankova et al. [23] reported that SCFA as epigenetic regulators might affect genes assumed to be involved in ASD. BA and PA were able to increase catecholamine production as HDACI by regulating the tyrosine hydroxylase (TH) gene in an in vitro neuronal cell line (PC12 cells). PA and BA also modulated lipid homeostasis and inflammatory processes [23]. Moreover, SCFAs’ influence on various genes of the dopaminergic pathway were detected, specifically on dopamine beta-hydroxylase (DBH) which, when dysregulated, shows associations with ASD in humans [23]. Interestingly, the serotonin system has been shown to only be affected by the administration of PA [52]. Furthermore, the study presented downregulating effects by PA or BA in the expression of fragile X mental retardation 1 (FMR1), neurexin and neuroligin, genes previously reported to relate to ASD [53,54,55,56]. BA, among all SCFAs, is the most important HDACI to modulate brain function through epigenetic processes [57] and thus, altered BA levels might potentially modify neuronal function. 

In addition to the potential role of BA and PA in ASD, it is worth noting that the structurally related valproic acid (VPA), a branched SCFA, effectively creates a frequently used ASD mouse model that mimics both behavioural as well as gut microbiome traits in ASD patients [58]. In addition, prenatal exposure to VPA significantly increases the risk of ASD and showed epigenetic effects on neurotransmitter homeostasis via HDACI, similarly to BA and PA [59,60,61,62]. Additionally, VPA invokes dysfunctions in glutamate and GABA-neurotransmission and is thus likely to produce an altered balance between excitation and inhibition in the cerebral cortex [63].

These studies have shown that alterations of SCFAs can intricately influence neurodevelopmental processes via epigenetic modulation as HDACI. In support of a connection to ASD, Stilling et al. [64] detected upregulated cAMP response element-binding protein (CREB)-dependent gene expression in amygdala of GF mice, a limbic structure involved in emotion, memory and behaviour. It is thus understandable that a dysfunctional amygdala has been associated with neuropsychiatric disorders like anxiety disorder, post-traumatic stress disorder (PTSD) and ASD [65].

In contradiction with the above data, no significant changes in SCFA production were found in GF mice inoculated with microbiota from poor growth and good growth preterm infants, even though the administered microbiota was associated with pathologic developmental changes in neurons and oligodendrocytes of the receiving mice [66]. This might point to a different and/or additional pathway than SCFA, by which gut microbiota may affect early neurodevelopment.

Overall, support for SCFAs as putative influencers on ASD are present in a handful of clinical and mainly preclinical studies, though the research is still in its infancy. Therefore, further investigation to bring light into this emerging theory is strongly recommended. 

### 3.2. SCFAs and Affective Disorders

Pathophysiological factors in affective disorders are multifaceted and gut microbial involvement has gradually become a potential contributing factor. Faecal SCFA levels from humans [67,68] and primates [67,68] with major depressive disorder (MDD) showed an overall decrease and altered composition compared to HCs (Table 1). AA, PA and isovaleric acid significantly decreased while only isocaproic acid increased in faecal samples of depressed individuals [67,68]. In contrast, one study reported no significant changes in faecal SCFAs in depressed patients [69]. Nevertheless, researchers previously showed distinct differences between faecal microbial compositions of HCs and MDD through taxonomic association studies [70]. Further links between affective disorders and a disturbed gut environment might be provided through observations in functional gut disorders like irritable bowel syndrome (IBS), exemplified by the results of a recent meta-analysis with significantly increased anxiety and depression in IBS patients [71]. 

A mentionable study by Kelly et al. [69] presented that depressive behaviour can be transferred from humans to germ-free rats by FMT, suggesting a strong connection between gut bacteria and affective disorders like major depressive disorder (MDD). Interestingly, there were discrepant findings regarding the role of SCFAs: faecal AA and total SCFA levels were higher in rats receiving FMT from patients than from HCs. However, depressed and healthy human donors showed no significant differences in their faecal SCFA levels. This calls for further investigations in clinical studies since interspecies differences might be a contributing factor in this case. Recently, rats bred for high anxiety-like behaviour (HAB), an animal model for anxiety and depression, displayed lower microglia numbers in distinct brain regions (infralimbic and prelimbic prefrontal cortex) and gut microbial shifts toward decreased counts of the BA-producing Lachnospiraceae family [72]. Treatment with antibiotic minocycline alleviated male HAB rats of depressive symptoms, further decreased circulating inflammatory cytokines and microglial count, as well as enriched their microbiota with known BA and 3-OH-butyrate producers Lachnospiraceae and Clostridiales family XIII. In fact, Clostridia are considered as the main BA-producing class of the human gut microbiome [73] (Table 2). These findings, together with previous propositions for immunomodulatory effects of BA and 3-OH-butyrate on inflammation, T-cell and microglial activity [13,28,29,74,75] point towards an intricate relationship between microbial derived SCFAs and affective disorders, that might benefit from their anti-inflammatory effects. In support of this theory, increased markers of inflammation such as pro-inflammatory cytokines in circulation and the brain are correlated with MDD [76]. Moreover, studies have successfully demonstrated SCFA-mediated anxiolytic and antidepressant effects in mice undergoing induced psychosocial stress [77]. In particular, the administration of sodium butyrate (NaB, the sodium salt of BA) has been reported to alleviate pathologic affective behaviours in rat models, including hyperactivity, depressive and manic symptoms [26]. Future work on this subject, especially through metabolomic studies in humans, might enlighten the intricate gut bacterial metabolite–brain axis interplay in affective disorders, as the current state of research provides only few clinical studies on this particular subject.

### 3.3. SCFAs and Autoimmune Diseases of the Brain: Multiple Sclerosis (MS)

Multiple sclerosis (MS) is an autoimmune disease of the CNS that mainly damages the myelin sheaths of motor neurons. An imbalance between anti-inflammatory Treg cells and proinflammatory Th1 and Th17 cells are widely understood to take part in the MS pathophysiology [78].

Individuals with MS have been reported to harbour microbiomes that are significantly different from HCs [79,80]. Indeed, one recent study reported increased *Streptococcus,* decreased *Prevotella_9* and overall decreased faecal SCFAs (AA, PA and BA) in a Chinese cohort of MS patients [81]. *Streptococcus* is known to produce all SCFAs [44,82] and *Prevotella_9* is able to generate AA and PA [81] (Table 2). MS patients displayed higher abundance of inflammatory Th17 cells, as anti-inflammatory Treg cells were decreased. Interestingly, faecal SCFA concentrations positively correlated with levels of circulating Treg cells in this study, thus suggesting that SCFAs exert anti-inflammatory effects due to elevated Treg/Th17–cell ratios. Similarly, significantly decreased SCFAs—were detected in blood samples of patients with active secondary progressive MS [29]. These two human studies might suggest an overall decrease in faecal and consecutively depleted circulating SCFA levels in MS patients (Table 1), that might shift the immune system towards proinflammatory processes due to lower Treg/Th17 cell ratios. 

Autoimmune processes in the CNS were affected by the gut through SCFAs and long-chain fatty acids (LCFAs) in the experimental autoimmune encephalomyelitis (EAE) mouse model of MS. The differentiation of pro-inflammatory Th1 and Th17 cells were increased by LCFAs, while anti-inflammatory Treg cell differentiation was boosted by SCFAs through the downregulation of the JNK1 and p38 pathway. Therefore, LCFAs exacerbated, while SCFAs alleviated disease and subdued axonal damage. Additionally, PA demonstrated the most stimulating effect on Treg cell differentiation, which improved histopathological outcomes of the spinal cord in EAE mice [13]. Melbye et al. [83] reviewed two other studies in EAE mice, who supported the ameliorating role of SCFAs in disease activity by modulating an increase in anti-inflammatory Treg cells and a decrease in pro-inflammatory Th1 and Th17 cells. BA too, was able to ameliorate demyelination in rats and importantly, exposing an organotypic slice culture to BA resulted in suppressed lysolecithin-induced demyelination and enhanced remyelination, represented by higher counts of mature oligodendrocytes [84]. In congruence with these studies, a recent review concluded that PA and BA ameliorated the clinical symptoms of EAE by inducing immune tolerance epigenetically as HDACIs. The proposed mechanism involves an upregulation of the transcription factor Foxp3 leading to increased Foxp3+ T regulatory lymphocytes, also known as Treg cells that inhibit proinflammatory Th1 and Th17 cells [85]. In addition to these findings that support an overall anti-inflammatory effect through SCFAs, Park et al. [29] recently demonstrated that SCFA administration to EAE mice models increased anti-inflammatory IL10+Tcells and IL-10, as well as pro-inflammatory Th1, Th17 and Tc cells. Moreover, SCFA receptors GPR41 and GPR43 have demonstrated proinflammatory effects in EAE pathogenesis [29]. These results underline the importance of SCFAs to protect from inflammatory processes in the CNS. Their uncovered pro-inflammatory effects, however, indicate a complex system in immunomodulation, which calls for further work in this subject in order to evaluate potential interventions involving SCFAs in neuroinflammatory diseases.

### 3.4. SCFAs and Neurodegenerative Diseases of the Brain

Neurodegenerative diseases are becoming increasingly prevalent as the population gradually grows older. Researchers are trying to elucidate the pathomechanisms of the various brain diseases including Alzheimer’s disease (AD), Parkinson’s disease (PD), dementia with Lewy bodies (DLB), multiple system atrophy (MSA) and Huntington’s disease (HD) [86]. This chapter will first briefly list some findings on SCFAs and neurodegenerative processes in general before focusing on AD and PD.

#### 3.4.1. General Findings on Neurodegenerative Processes

A recent in vitro study investigated the direct influences of the SCFAs NaB, sodium valerate and hexanoic acid on neuroinflammation and found that high concentrations of NaB were able to decrease the basal levels of the proinflammatory cytokine IL-6 in human glioblastoma–astrocytoma U373 cells [87]. However, further findings showed no neuroprotection from induced oxidative stress in differentiated SH-SY5Y cells (human-derived neuroblastoma cells) by any SCFAs. Interestingly, exposure to BA and valerate was able to induce neuronal maturation through MAP2-gene expression in undifferentiated neuroblastoma cells, thus hinting towards a beneficial effect on neurogenesis [87]. BA’s effects in animal models include the potential to alleviate impaired cognition, enhancing neuronal plasticity, improve learning and memory performance, as well as neuroprotection, all beneficial processes regarding neurodegenerative diseases [57].

Overall, direct impacts on brain cells by SCFAs seem to be complex as well as dose-dependent, which supports a hypothesis that anti-inflammatory processes in the brain, neuroplasticity and neurogenesis could be positively modulated through the manipulation of gut bacterial production and/or external supplementation of SCFAs. Recent research further provided evidence for an ameliorating role of SCFAs in inflammatory hippocampal neurodegeneration in mice through the reduced impairment of the intestinal barrier, which was induced by a high-fructose diet. It was suggested that SCFAs could amend the faulty colonic NLRP6 inflammasome responsible for epithelial impairment to alleviate hippocampal neuroinflammation, thus possibly reducing the likelihood of neurodegenerative processes associated with a typically high-fructose Western-style diet [88]. This might be an indirect mechanism by which SCFAs could exert neuroprotective effects.

#### 3.4.2. SCFAs and Alzheimer’s Disease

Gut microbiome of Alzheimer’s disease (AD) patients were observed to be altered, with decreased overall richness and diversity as well as some shifts within taxonomical compositions [89,90]. Some studies presented AD progression to associate with dysbiosis and that a healthy gut microbiome provides beneficial effects in AD patients and rodent models [90,91]. Recent studies further showed significant changes in gut microbiome compositions between AD patients and HCs at the genetic level, suggesting some bacterial AD-associated PCR products to be a potential marker of AD risk [92]. As Franceschi et al. described in their review in 2019, disturbances in the gut microbiome might influence processes involved in AD pathogenesis, such as chronic inflammation, molecular mimicry and Aβ accumulation. Furthermore, the presence of microbiome enterotype III (low *Bacteroides* and *Prevotella*) and the absence of enterotype I (>30% *Bacteroides*) were reported with stronger associations to the presence of dementia than classic markers (Table 3) [93]. This highlights the potential of the GBA to impact pathogenesis in dementia, though unfortunately, no human studies that measured SCFA faecal levels have been reported as to our literature search.

The GF condition in transgenic AD mice models were observed to slow the progression of disease symptoms [94], underlining an important role for the presence of the gut microbiome, including bacteria and their metabolites in AD pathogenesis. A study with the APP/PS1 mouse model of AD reported disturbed microbiota composition and diversity, as well as overall lower SCFAs levels compared to wild-type (WT) controls. Additionally, over 30 metabolic pathways possibly related to amyloid deposition and ultrastructural anomalies were detected in intestine samples of the AD group [95]. Zheng et al. [96] have introduced a method of stable isotope labelling and liquid chromatography–tandem mass spectrometry to sensitively detect 21 SCFAs in mice faecal samples of AD and WT mice. In an AD mouse model, decreased levels of PA, isobutyric acid, 3-hydroxybutyric acid, and 3-hydroxyisovaleric acid were detected while increased levels of lactic acid, 2-hydroxybutyric acid, 2-hydroxyisobutyric acid, levulinic acid and valproic acid were found. In contrast to these findings, faecal PA was enriched in mice receiving FMT from an AD donor in comparison to a healthy one [97]. However, two faecal donor samples selected out of groups of 14 healthy and 13 AD volunteers might limit that study’s evidential impact due to putative inter-individual variations.

The prevention of Aβ accumulation and the removal of accumulated amyloid plaque have been at the core of anti-AD therapeutic undertakings for more than two decades [98]. It is important to highlight an in vitro study reporting that valeric acid (VA), BA and PA, but not isobutyric acid, isovaleric acid and AA, to be capable of stopping the misfolding of Aβ40 peptides to neurotoxic Aβ40 aggregates in a dose-dependent manner [99]. Additionally, the same experiment on Aβ42 aggregation showed that only VA could inhibit the process dose-dependently. A third experiment determined that VA and BA successfully halted Aβ fibril formation in a dose-dependent manner. These results demonstrate a mechanism by which gut microbial-derived SCFAs may benefit AD patients and that a gut microbiome depleted of SCFA producers might promote neurotoxic amyloid build up in the CNS. In support of this theory, Sun et al. [100] reported that FMT from WT-mice to the APP/PS1 mice model of AD resulted in the alleviated brain deposition of Aβ as well as levels of neurotoxic Aβ40 and Aβ42, tau protein phosphorylation, synaptic dysfunction, neuroinflammation and cognitive deficits, accompanied with restored alterations in gut microbiota and faecal SCFA levels. The AD mice harboured a perturbed microbiome enriched with Proteobacteria, Verrucomicrobio (phylum level), and *Akkermansia*, *Desulfovibrio* (genus level), with depleted Bacteroidetes phyla. All these conditions were reversed through FMT treatment. However, these microbial changes were lacking consistency in the relative abundance of bacterial species, for example a relative increase in Bacteroidetes or BA-producing Firmicutes has been previously observed in animal and human studies of AD [91]. Therefore, definite conclusions about distinct AD gut microbiome compositions and their capacity of SCFA production cannot be made at this point in time, which further warrants our focus on disease correlations with bacterial metabolites instead.

Impaired epigenetic gene expression has been discussed as a key factor in AD pathogenesis [101], which conceivably led to a study of BA’s role as HDACI in an AD mouse model. Treatment with BA was able to improve associative memory function at an advanced stage of disease [102]. Other studies mentioned the neuroprotective capacity of BA to manipulate regulatory regions of the Forkhead box gene locus as HDACI. This provides a preventative and/or therapeutic potential to affect the balance between life-promoting and apoptotic cell processes critical in neurodegenerative diseases [8]. BA as NaB has shown neuroprotective benefits as HDACI in studies of PD, AD and HD, particularly leading to improved learning and memory in dementia, the prevention of oxidative stress and neuronal cell death in HD and PD, as well as overall upregulated transcription of neurotrophic factors involved in plasticity, survival and regeneration [103]. These results might indicate that decreased or overall altered gut microbial SCFAs and thus, dysregulated histone-acetylation, might indeed be connected to AD and related brain diseases. We therefore suggest future studies to look for putative impacts of altered SCFA-producing gut microbiota on AD-related epigenetic processes in the brain. SCFAs might also impact AD indirectly through additional pathways via the regulation of intestinal gluconeogenesis by FFAR3 signalling, which affects the activity of the dorsal motor nucleus of the vagus, a structure with altered activity in PD and AD [8]. BA especially has also been hypothesized to positively impact cognition in AD patients via the stimulation of vagal afferents [8].

In a study with rats fed a high-fat diet, it was shown that the administration of two valeric acid esters (monovalerin and trivalerin) led to higher levels of AA in the brain, serum and liver, while caecal levels decreased. These data suggest that AA can actually be increased in the brain by oral supplementation and uptake in the gut [104]. This might be of interest, since AA administration to lipopolysaccharide (LPS)-stimulated astrocyte cultures was successful in producing anti-inflammatory effects [105]. BA exposure invoked anti-inflammatory effects as well, as shown by the reduced microglial activation and decreased secretion of inflammatory cytokines. BA inhibits the secretion of HDAC gut microbe-derived circulating inflammatory cytokines and thus limits their effects on neuroinflammatory processes that have been postulated to be involved in AD pathology [91]. Pro-inflammatory cytokines derived from dysbiosis might invoke the formation of Aβ aggregates as well as cause the dysfunctional maturation of microglia, thus leading to increased amyloid accumulation in the CNS [91]. Taken together, healthy gut flora with undisturbed SCFA production might benefit AD patients with decreased neuroinflammation and amyloid accumulation. 

#### 3.4.3. SCFAs and Parkinson’s Disease

PD is, after AD, the second-most prevalent neurodegenerative disease in the world [106] and is part of a cluster of neurodegenerative disorders associated with aggregated amyloid proteins. Misfolded alpha-synuclein proteins (αSyn) are specifically implicated in PD, DLB and MSA, also jointly known as “Synucleopathies” [107]. In PD, the dopaminergic neurons residing in the substantia nigra pars compacta are lost, subsequently leading to impaired motor functions [108]. Gut dysbiosis and GI dysfunction have been repeatedly mentioned as a hallmark of PD [108,109,110,111,112,113,114], thus investigations of mechanistic processes involving the GBA have emerged in recent years. This conceivably led to questions about gut microbial participation in pathophysiological processes of PD, such as the spreading of αSyn aggregates from gut to brain via the vagal nerve [115] as well as probable connections between gut dysbiosis, neuroinflammation and misfolding of αSyn [116].

Overall decreased SCFA levels with relatively low BA and a microbiome with reduced Bacteroidetes, Prevotellaceae as well as enriched Enterobacteriaceae were reported in PD [117] (Table 1). Underlining these findings, a recent review reported trends of reduction in SCFA producers in a PD patient’s microbiomes, specifically reduced Lachnospiraceae (*Blautia*, *Dorea*, *Coprococcus*, *Rosburia*, *Clostridium XIV*), *Faecalibacterium* and *Bacteroides* [109]. Interesting to mention is the overall increased abundance of Enterobacteriacea, a phylum that is known to produce SCFAs (Table 2) and to also associate with the severity of motor symptoms in PD patients [112]. This finding might at the first glance appear counterintuitive under the assumption that SCFAs and their producers are beneficial to PD. On the other hand, the relative abundance in Enterobacteriaceae might further indicate the production of other metabolites involved in PD, as will be elaborated on later in the chapter discussing bacterial amyloids.

Two studies in rodents reported further contradicting results regarding SCFA levels in PD. Sampson et al. [118] used a transgenic αSyn-overexpressing mouse model of PD, that presented ameliorated PD pathologies when in a germ-free (GF) state or treated with antibiotics (AT). These GF/AT mice were then inoculated with human PD-donor microbiota. This treatment significantly altered faecal microbial communities and SCFA composition, displaying lower AA, but higher PA and BA, as well as worsened motor dysfunction compared to those receiving healthy FMT. Thus, the administration of a mixture of SCFAs to GF/AT mice was effective in inducing motor deficits, as well as αSyn aggregation and microglial activation in the brain. This suggested a relevant role for SCFAs as mediators of PD in a genetically susceptible animal model [118]. Supporting these findings, the 1-methyl-4-phenyl-1,2,3,6-tetrahydropyridine(MPTP) – induced PD mice model presented an increased abundance of faecal SCFAs. The gut microbiome of this PD model was administrated to normal mice, which resulted in motor impairment and decreased striatal neurotransmitters, while FMT from healthy donors alleviated those symptoms [119]. The inconsistencies within the previously mentioned studies in human subjects, regarding beneficial or detrimental effects of SCFAs in PD, might point towards inter-species differences of mice and humans and the GF state of acutely inoculated mice. This could further suggest that even though the presence of SCFAs seems necessary to trigger pathological changes in genetically vulnerable organisms, shifts towards depleted SCFA levels and their bacterial producers might play a role in already established PD.

Several lines of evidence suggest that SCFAs, BA in particular, may exert possible beneficial effects in PD. First, BA might play a role in PD as a neuroprotective agent due to its agonistic effect on the receptor GPR109A, which promotes anti-inflammatory processes [120]. In addition, BA might also benefit PD patients with reduced neuroinflammation, indirectly enhanced dopamine synthesis through increased free niacin levels, as well as improved energy homeostasis and mitochondrial function [103,121]. Lastly, SCFAs ameliorated dysfunctional microglia in GF mice, which was represented by improved microglial maturation, morphology and function [28]. Proper mature microglial function includes decreased inflammatory activity and phagocytosis for amyloid proteins like tau, Aβ, and αSyn. Therefore, the state of the gut microbiome and its production power for SCFAs might positively influence several aspects of neurodegenerative diseases [91]. It might be of interest that mice lacking the SCFA receptor FFAR2 have shown dysfunctional microglia similar to GF animals, however, that particular study suggested alternative pathways by which SCFAs directly exert their effects on microglia due to a lack of evidence for FFAR2-expression on CNS cells [28]. Definite mechanisms involved in receptor-mediated processes of SCFAs remain to be determined.

As previously mentioned, SCFAs can upregulate gene-expression as HDACI. This process was shown to facilitate neuroplasticity and long-term memory, involving CREB-dependent gene regulation [122,123]. In vitro studies also discovered PA and BA to modulate transcription of the tyrosine hydroxylase gene in brain cells and thus to influence catecholaminergic biosynthesis [23]. Catecholamines like DOPA, dopamine (DA), noradrenaline and adrenaline are essential neurotransmitters with important roles in brain diseases, exemplified by the depletion of DA being a key factor in PD [52,106,124]. Especially relevant to PD is that the enzyme tyrosine hydroxylase catalyses the rate-limiting step of DA synthesis [8]. Further research on BA’s role as HDACI revealed protective effects for dopaminergic cells, namely rescuing them from αSyn-mediated DNA damage [125] or MPP+-induced toxicity [126] through an enhanced expression of DNA damage response genes. Supporting evidence come from a study in a drosophila model of PD in which BA has been reported to alleviate motor dysfunction and mortality [127]. Moreover, altered gut levels of SCFAs and neurotransmitters were associated with the surface area of the insula [9], a brain region that is understood to be dysfunctional in neurological and psychiatric disorders [128]. 

The influence of gut microbiota on PD might further impact the conventional therapy of levodopa administration, since the abundance of the gene for tyrosine decarboxylase, an enzyme converting levodopa to DA, in the microbiome of PD patients correlates with higher dosage needs for levodopa/carbidopa. Furthermore, it was shown in rats, serum levels of the aforementioned drug negatively correlated with the host’s microbiome tyrosine decarboxylase gene levels [129]. These findings might provide the base for further clinical studies on gut microbial modulations in PD patients with increased levodopa/carbidopa dosages.

Taken together, SCFAs seem to exert overall beneficial effects on the CNS regarding autoimmune brain diseases and neurodegenerative diseases. However, preclinical findings on probable detrimental effects upon SCFA exposure in rodents suggests that these bacterial metabolites might function as double-edged swords when it comes to brain health. Thus, the thorough examination of these mechanisms is crucial before future potential therapeutic and preventative strategies can be unequivocally suggested.

## 4. Non-SCFA Bacterial Metabolites

The vast majority of current studies on the GBA involve SCFAs. Our gut microbiota, however, produces metabolites far beyond the products of fibre degradation, including vitamins, polyphenol metabolites and products from amino acid metabolism (Figure 1). Each of these families of compounds are involved in various pathways and contain potential neuroactive metabolites [22]. This warrants our curiosity in exploring non-SCFA bacterial metabolites as contributors to the GBA.

### 4.1. Amino Acid Metabolites

Metagenomic studies suggests human gut microbes to be largely involved in amino acid metabolism [133]. Of special interest are the aromatic amino acids (AAA) tyrosine (Tyr), phenylalanine (Phe) and tryptophan (Trp). Humans are unable to produce AAA and depend on dietary sources and our gut microbiome for covering their nutritional needs. Gut bacteria are able to synthesize all three AAA de novo via the shikimate pathway [92,134]. In a first step, Trp and Phe are biosynthesized. Tyr is then synthetized from Phe. Further AAA metabolism occurs in the host as well as in gut microbes like *Lactobacillus*, Enterobacteriaceae and anaerobes of the phylum Firmicutes, that generate other metabolites. Phe and Tyr are catabolized in animals to neurotransmitters, including L-Dopa, DA, epinephrine and norepinephrine, while gut bacteria are able to produce phenolic compounds like p-cresol from Tyr and phenyl molecules from Phe. Trp is an essential precursor for the neurotransmitters serotonin and tryptamine, as well as vitamin B3 (niacin), redox cofactors NAD(P)+, plus metabolites from the kynurenine pathway [134]. On note, the kynurenine pathway in gut microbes generate metabolites associated with brain functions like indole, indole-derivatives, kynuric acid and quinolinate, which will be elaborated on in the following chapters. For a more in-depth analysis of AAA metabolism in plants, microbes as well as mammals, we refer the reader to the extensive review by Parthasarathy et al. [134].

**Figure 3:**
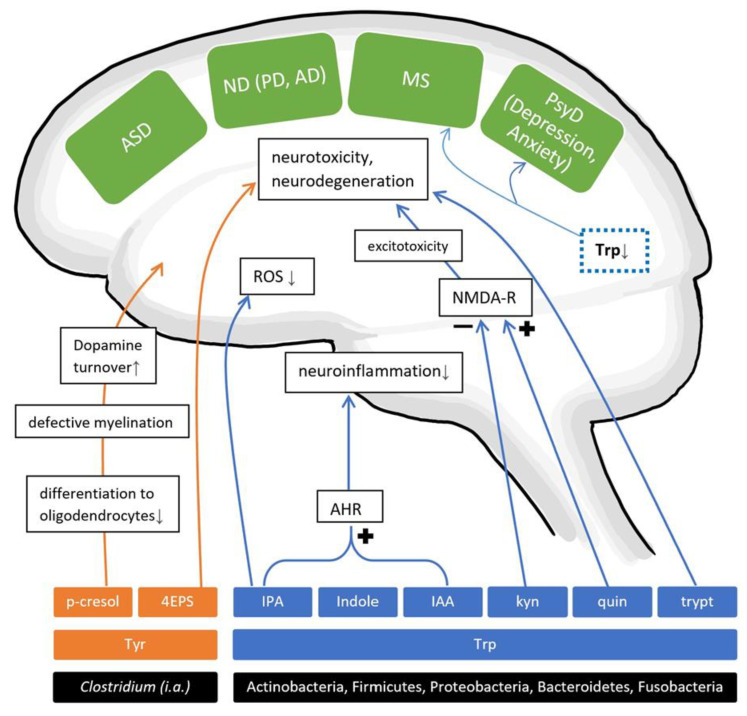
Hypothetical influences on brain diseases by gut bacteria-derived tyrosine and tryptophane metabolites. This figure illustrates the mechanistic effects by which gut microbial metabolites might influence brain functions related to autism spectrum disorder (ASD) and neurodegenerative disorders (NDs): Parkinson’s disease (PD), Alzheimer’s disease (AD), multiple sclerosis (MS) and psychiatric disorders (PsyD). Gut bacteria taking part in metabolite production are listed in black boxes situated under the orange (tyrosine metabolites) and blue boxes (tryptophane metabolites). Arrows accompanied with + or − represent an agonistic (+) or antagonistic (−) effect on a receptor, whereas unaccompanied arrows symbolize an effect described in the white boxes. ↑ = upregulated, ↓ = downregulated or lowered levels of. ROS = reactive oxygen species; NMDA-R = N-methyl-D-aspartate receptor; Trp = tryptophane; AHR = aryl hydrocarbon receptor; 4EPS = 4-ethylphenylsulfate; IPA = indole-propionic acid; IAA = indole-acetic acid; kyn = kynurenine; quin = quino-linate; trypt = tryptamine; i.a. = inter alia.

Considering the previously described processes in AAA metabolism, it is conceivable to assume that gut microbiota might modulate neurotransmitter metabolism, synthesis, and availability in the gut, the circulatory system and the CNS. In fact, the abundance of circulating Trp can be curbed as a result of gut microbial Trp metabolization through other pathways, thereby possibly limiting the precursor for neurotransmitter synthesis in the CNS while also generating other neuroactive metabolites like indole and its derivatives [135] (Figure 3). On the other hand, gut microbes seem to elevate serotonin plasma availability after colonizing GF animals, leading to the assumption that the presence of a functioning gut microbiome contributes to physiological serotonin plasma levels [136]. More importantly, a recent study observed gut microbial involvements in Trp metabolism, providing an extensive overview of six pathways, each generating neuroactive metabolites referred to as “TRYP-6”, consisting of kynurenine, quinolinate, indole, indole acetic acid (IAA), indole propionic acid (IPA) and tryptamine [135]. They identified five common gut-inhabiting phyla capable of two to six pathways. The five phyla Actinobacteria, Firmicutes, Proteobacteria, Bacteroidetes and Fusobacteria thus have been suggested to relevantly influence Trp metabolism. Investigations on a genus level revealed that *Clostridium*, *Burkholderia*, *Pseudomonas*, *Streptomyces* and *Bacillus* were particularly capable of generating neuroactive Trp metabolites, with the first two holding the highest potential (Table 4). Numerous pathways and metabolites in the AAA metabolism, especially Trp, show relevant effects on the CNS that seem to be intricately complex and crucial for proper brain function, thus pointing to these non-SCFAs as promising players on the GBA (Figure 3).

#### 4.1.1. AAMs and Neurodevelopmental Disorders

P-cresol is a known uremic toxin, which is metabolized into p-cresol sulphate by the liver [137] and is believed to derive from Tyr fermentation in several gut bacterial species (Table 4). Significantly increased urinary and faecal levels of p-cresol were reported in autistic children, with some linking urinary levels with the clinical severity of disease [39,41,138,139]. Interestingly, p-cresol levels significantly and negatively correlate with age in ASD patients, which might suggest that younger individuals with ASD are exposed to effects from elevated p-cresol levels [41]. One study, however, did not detect significantly altered faecal levels in children with ASD [42]. As for non-human studies, p-cresol was very recently shown to dose-dependently induce and exacerbate ASD-like behaviours and significantly activate dopamine (DA) turnover in brain regions (amygdala, nucleus accumbens and striatum) in the genetically vulnerable BTBR mice model for ASD [140]. Social avoidance behaviour and increased gut levels of p-cresol were detected in GF mice, inoculated with p-cresol-producing Clostridiales (including Lachnospiraceae and Ruminococcae families), and these mice associated with defective myelination in the prefrontal cortex [141]. Additional in vitro testing showed that exposure to p-cresol interrupted the differentiation of progenitor cells into oligodendrocytes [141], suggesting that gut microbial p-cresol might impact CNS myelination through transcriptional changes. Other mechanisms by which p-cresol might negatively impact neuronal functions [140] include the inhibition of dopamine-β-hydroxylase and membrane depolarization with higher vulnerability for seizures and blunted Na+/K+-ATPase function. These mechanisms might demonstrate a potential for gut bacterial-derived p-cresol to play a role in disorders with disfunctions in the CNS, including ASD, MS, and neurodegenerative diseases.

In the maternal immune-activated (MIA) mouse model of autism spectrum disorder, changes in serum metabolites, showing significant elevations of two AAA bacterial metabolites 4-ethylphenylsulfate (4EPS) and indolepyruvate were found, which were completely normalized along with ASD-related behaviour, dysbiosis and impaired gut barrier after the inoculation with the probiotic *B. fragilis* [142]. Moreover, WT mice treated with the metabolite 4EPS alone manifested anxiety-like behaviour similar to MIA-mice, thus suggesting a compelling association between 4EPS and ASD. Additionally, other metabolites, two of them being serotonin and p-cresol, were elevated in the serum of MIA-mice, though not at significant levels [142]. It is essential to mention 4EPS’s structural similarity to the prior mentioned p-cresol, which has links to ASD and is believed to share its producers in the gut with 4EPS, namely *Clostridia* spp. [36,140,142] (Table 4). Overall, preclinical data and some supportive human studies point towards a connection between 4EPS, indolepyruvate, p-cresol and ASD, not only as biomarkers of disease but also as putative mediators of pathogenesis.

With an extensive in silico study, Kaur et al. [135] recently detected the aforementioned “TRYP6”, the six Trp metabolism pathways generating neuroactive metabolites, to be enriched in the metagenome of autistic gut microbiota. Genomes of the genera *Burkholderia* and *Pseudomonas* showed particularly large potentials for TRYP6 metabolism. *Burkholderia* holds pathways for kynurenine and quinolinate, with a lower production of IAA, indole and tryptamine, while *Pseudomonas* is a strong producer for kynurenine and a weaker one for IAA, quinolinate and tryptamine. Other enriched pathways in autistic children consisted of those generating indole and its derivative IAA by already mentioned genera *Burkholderia* and *Pseudomonas* plus *Corynebacterium*. Though microbiota from NT individuals also harboured some relatively enriched bacteria capable of producing TRYP6, namely *Alistipes* for indole and *Eggerthella* for IAA production, these genera are comparatively weak producers and thus, theorized to use indole and IAA as inter-bacterial communication tools [135]. Similarly, altered Trp metabolism in ASD has been indicated through significantly increased urinary levels of IAA, indoxyl sulphate (IS, also known as indican) and indolyl lactate in autistic children [143], though to date no data on faecal levels have been found.

In addition to Trp metabolism, altered Phe metabolism in autistic children were recently highlighted, partly based on evidence of significantly elevated Clostridia-generated Phe metabolites in the urinary profiles of ASD patients including 3-(3-hydroxyphenyl)-3-hydroxypropionic acid, 3-hydroxyphenylacetic acid and 3-hydroxyhippuric acid [36]. However, whether these metabolites might be modulated by gut bacteria remains to be elucidated.

Increased faecal levels of glutamate in children with ASD, as well as decreased GABA levels in those with pervasive developmental disorder not otherwise specified (PDD-NOS) have been previously reported [39]. Kang et al. [41] have similarly detected lower GABA faecal levels in autistic children, though these did not reach significance (*p* = 0.077). Preclinical observations in altered GABA and glutamate levels were made in a study invoking behaviours associated with ASD in GF mice through inoculation with gut microbiota derived from autistic patients. In comparison, mice receiving healthy donor FMT did not produce ASD-like behaviour [45]. Moreover, lower faecal levels of the GABA A receptor agonists 5AV and taurine were found in the first group, supporting a putative role of disturbed GABA signalling in ASD. In a further step, the exposure of an ASD mouse model to taurine or 5AV during the prenatal and weaning period produced mice with ameliorated ASD behaviour in comparison with mice treated during their juvenile stage and older mice. This suggests a critical window of vulnerability for disturbed GABA signalling during neurodevelopment [45]. Other researchers were able to uncover correlations between gut microbe genes associated with neurotransmitter metabolism and the surface area of the insula, with a focus on two microbial genes involved in GABA and glutamate metabolism, namely 4-hydroxybutyrate dehydrogenase and glutamate dehydrogenase [9]. As already mentioned, the insula is thought to be dysfunctional in many psychiatric disorders with disturbed emotion, cognition and motivation, such as affective, neurodevelopmental and neurodegenerative disorders [128]. However, it is also important to note that Kang et al. [41] were not able to detect any significant changes in gut bacterial pathways by PICRUSt database analysis between ASD and NT children. Nevertheless, these studies accumulatively show the potential involvement of gut microbial metabolites in disturbed GABA and glutamate signalling in ASD pathophysiology. Further metabolomic, metagenomic and microbial analyses of faecal amino acid metabolites (AAMs) are nonetheless highly encouraged. Compellingly, a recent study reported that GABA produced from gut bacteria (*E. coli HT115* and *P. aeruginosa PAO1*) was able to protect from neurodegeneration in the nematode C. elegans [144].

Lastly, the amplified metabolism of the amino acids Tyr, lysine, cysteine and methionine in healthy children’s gut microbiomes have been found, which implies a supportive function of gut commensals during brain development, since the mentioned amino acids are not only substrates for the synthesis of structural proteins, but also neurotransmitters and biogenic amines [145].

#### 4.1.2. AAMs and Psychiatric Disorders

No specific studies on non-SCFA faecal metabolites have been found in humans with affective disorders. However, there are a handful of reports pointing to correlations between disturbed gut microbial Trp metabolism and psychiatric disorders like anxiety and depression. Studies on acute tryptophan depletion (ATD) in humans demonstrated a correlation between the reduced levels of circulating Trp and depressive symptoms in patients, who are responsive to treatment with selective serotonin reuptake inhibitors [146,147]. ATD has also been shown to worsen depressive symptoms in patients in remission, as well as in healthy volunteers at high risk for depression [70]. Furthermore, 5-HT levels in the CNS were shown to be impacted by the amount of dietary Trp in humans [147]. This raises the question of whether a disturbed gut microbial Trp metabolism could deplete circulating Trp availability and consecutively impact 5-HT homeostasis in the CNS. The impact of gut microbiota, or the lack thereof, on the host’s nervous system can be explored in GF raised animals providing the evidence for altered levels of neurotransmitters in the brain in comparison to conventionally raised control animals [148]. In support of the previous reports on humans, a recent study in GF mice showed initially higher Trp and 5-HT brain levels together with a less depressive behaviour at baseline and intriguingly, decreased Trp and 5-HT with enhanced depressive behaviour after ATD compared to the control group (specific pathogen-free mice) [149]. Additional support was obtained by findings in a rat model, which showed an induced depression to invoke gut microbial alterations as well as noticeable faecal metabolite shifts [150]. Sixteen metabolites were evaluated to be significantly distinct enough to function as depression biomarkers, including altered Trp metabolites (upregulated dextrorphan O glucuronide, 3-methyldioxyindole and downregulated 5-methoxytryptophan) (Table 5). The others consisted of bile acid metabolites (upregulated) as well as hypoxanthine (upregulated) and fatty acid metabolites (downregulated). Additionally, the altered gut microbiota also resulted in changes of catecholamine levels in the hippocampus of depressed rats, specifically serotonin (5-HT) and DA [150].

Similarly, Clarke et al. [151] found elevated levels of 5-HT and 5-HIAA in hippocampal structures of GF male mice, as well as higher plasma levels of their precursor Trp. Considering that CNS Trp levels are to a great extent regulated by its abundance in plasma, this supports the conjecture of a humoral pathway through which gut microbes could influence serotoninergic neurotransmission by modulating Trp availability [148]. Fascinatingly, it was not possible to reinstate altered hippocampal 5-HT levels through inoculation with an intestinal microbiota in GF mice at a later stage of life, even though the serum levels of Trp were normalized. This points to a critical time window in which microbial Trp metabolism could directly impact neurodevelopment [152]. Additionally, GF animals displayed elevated stress reactivity, represented with higher corticosterone production, while also expressing lower anxiety-like behaviour that was normalized after recolonization [148]. This is intriguing, since stress hormones like cortisone shift Trp metabolism away from 5-HT production to the kynurenine pathway that generates kynurenic acid, quinolinic acid and picolinic acid [153]. While kynurenic acid invokes antagonistic effects on the α7 nicotinic acetylcholine receptor and the N-methyl-D-aspartate (NMDA) receptor, quinolinic as well as picolinic acids are agonists of the NMDA receptor with neurotoxic and depression-producing properties [152]. It is also noteworthy, that a previously mentioned study on ASD reported the induction of anxiety-like behaviour in mice following the administration of the microbial Trp metabolite 4EPS [142]. Overall, these studies might provide tangible evidence for gut microbial impact on depressive and anxious behaviour by regulating the availability of circulating Trp and consecutively, levels of Trp metabolites like 5-HT, kynurenic, quinolinic and picolinic acids. However, clear associations between gut microbial Trp metabolites and depression or anxiety seem too early to be made since studies on this subject are largely based on preclinical settings on animals.

Indole is partly produced from dietary tryptophan through the enzyme tryptophanase [154] mainly by gut bacteria *Escherichia*, *Citrobacter*, *Fusobacterium*, *Bacteroides*, *Clostridium_XIX*, *Desulfitobacterium*, *Edwardsiella*, *Providencia* and *Shigella* [135] (Table 4). Indole has also recently been shown to be associated with impaired motor activity, anxiety and depression in rats when acutely or chronically overproduced [155]. Probable pathways by which these effects occur are the activation of vagal afferences by indole and on the other hand, accumulation of oxidized indole derivatives like oxindole and isatin in the brain. Indole has been shown to activate gut mucosal L-cells to secrete glucagon-like peptide-1 (GLP-1), which then stimulates vagal afferent fibres, therefore presenting an indirect impact of indole on the CNS [156]. Oxindole is known to inhibit motor activity [155], invoke hypotension, loss of righting reflex and a reversible comatose state, while isatin is proposed an anxiogenic role by inhibiting monoamine oxidase (MAO) B and by producing antagonistic effects on benzodiazepine receptors in rodents [70]. However, Jaglin et al. [155] showed that while acute overexposure to indole in the rat gut produced depressant effects on motor activity and elevated levels of oxindole and isatin the brain, chronic exposure to indole-producing *E. coli* induced depression-associated traits (anxiety-like and helplessness behaviours) without an accumulation of oxindole and isatin in the CNS. This suggests that an indole-overproducing gut microbiome might be a risk factor for the development of anxiety and depression, while acute spikes of indole-production might profoundly decrease locomotion by the central accumulation of oxindole and isatin as well as activation of vagal afferences. Studies have further demonstrated vagal GBA connections to neurons related to reward centres, thus pointing towards a probable pathway for gut metabolites to influence the brain in neuropsychiatric disorders with disturbed reward systems [157]. A recent in silico study on microbial Trp metabolism pathways in neurological disorders called for further investigations of the gut microbiome in schizophrenia, since assumptions were too early to be made on one single available dataset, that showed altered indole, IAA and tryptamine pathways in the microbiome of schizophrenic patients [135]. The research of gut microbial influence on schizophrenia is still in its infancy, which is represented by very few analyses on microbiome compositions and no study on faecal metabolomes in schizophrenic cohorts so far. With the emerging correlations between other brain disorders and the microbiome, as well as some preclinical information on dysbiosis and probiotic studies in schizophrenia [158], further work on this particular subject remains wanting of exploration. Taking all these studies into consideration, tryptophan metabolism with its manifold metabolites seem to be intricately influenced by gut microbial metabolites and to be implicated in psychiatric disorders and brain functions.

#### 4.1.3. AAMs and Neurodegenerative Diseases

##### Alzheimer’s Disease

A limited number of studies have indicated adverse effects on neurons in the context of Alzheimer’s disease (AD) by Trp metabolites. The decarboxylated molecule tryptamine has been associated with neurotoxicity and neurodegeneration [92,159,160,161] (Figure 2). Tryptamine producers commonly found in gut flora are *Holdemania*, *Desulfovibrio*, *Yersinia*, *Tyzzerella*, *Bacillus*, *Clostridium* and *Ruminococcus* [135] (Table 4). Most recent findings showed gut bacterial genomes in faecal samples of AD patients, of which one gene sequence encodes the enzyme Na-transporting NADH:Ubiquinone reductase (in *Clostridium sp.*), which produces the neuroprotectant ubiquinone. Interestingly, that enzyme is also involved in the metabolic synthesis of AAA [92]. Underlining these findings in AAA metabolism, Trp and Tyr (also GABA, taurine and valine) were found to be decreased in faecal samples of mice receiving FMT from an AD patient [97], though as noted in a previous chapter, two faecal donor samples selected out of groups of 14 healthy and 13 AD volunteers might limit the evidential impact by probable inter-individual variations.

Regarding the theory of a perturbed Trp metabolism in AD, microbial and hepatic enzymes generate kynurenine from Trp, and in succession, kynurenic acid or quinolinate. Quinolinate shows excitotoxic properties as an NMDA receptor agonist, whereas kynurenic acid ameliorates those neurotoxic effects as NMDA receptor antagonist. Therefore, this might provide a probable link between Trp metabolites and neurodegenerative processes in AD (Figure 3). In contrast to those findings, in aberrantly elevated amounts, kynurenic acid has been linked to cognitive impairments, probably caused by its antagonistic effect on the α7-nicotinic acetylcholine receptor [152]. It should be emphasized that CNS kynurenine mostly originate from the periphery and that its metabolization into kynurenic acid and quinolinate takes place in the CNS [151]. Some gut genera *Bacillus*, *Burkholderia*, *Streptomyces* and *Pseudomonas* are specially equipped for kynurenine production, while *Klebsiella*, *Bacillus* and *Burkholderia* are efficient quinolinate producers [135].

Another Trp metabolite generated from gut microbiota is indoxyl sulphate (IS), an uremic retention toxin in patients with chronic kidney disease, which has been associated with cognitive impairments [162] and various diseases of the brain such as AD, PD, MDD and MS [163]. IS was previously observed to induce nuclear factor-kappaB (NF-κB)-mediated oxidative stress in animal and in vitro studies [163,164]. Moreover, it exhibited potential neurotoxic effects in mice through perturbed microglial and astrocyte function, resulting in neuronal death [165]. This might be of interest since oxidative stress is proposed as a major process in neurodegenerative diseases [166]. Underscoring these correlations, researchers observed an elevated cerebrospinal fluid (CSF)/plasma ratio of IS in patients with PD compared to healthy counterparts [163]. This might suggest the increased crossing of IS through the BBB, a process probably facilitated by increased BBB permeability in diseases like AD and PD [167]. On the other hand, decreased IS levels in CSF, serum and faecal samples of GF and AT mice have been associated with perturbed fear extinction learning processes. These defects are common in anxiety and fear-related diseases with impaired learning and memory [168]. Considering these early preclinical and at times inconsistent findings on IS and brain disorders, future research focusing on microbiome-derived IS and its participation in neurodegenerative processes might enlighten this complicated and emerging subject. In a dementia-prone mice model, faecal metabolites seemed to differentiate from HC through higher levels of the amino acids ornithine and Tyr, which might excite further research, considering Tyr’s role as precursor for several crucial neurotransmitters (norepinephrine, epinephrine and DA) as well as for the uremic toxin p-cresol. Moreover, ornithine has shown protective effects in neurotoxic ammonia [114,169], though it remains to be determined whether bacterial metabolism is involved. Nevertheless, these reports overall suggest gut microbial modulated AAA metabolites as potential components of the complex and emerging field discussing the influence of GBA in AD.

##### Parkinson’s Disease

The analysis of gut microbial Trp metabolism in several databases of PD by Kaur et al. [135] detected enriched indole pathways and three of its producers to be differentially abundant in PD, namely *Alistipes*, *Akkermansia* and *Porphyromonas*. Concomitantly, enriched IAA production pathways in combination with increased *Lactobacillus* and *Staphylococcus* abundances were measured. Interestingly, distinct alterations of kynurenine and quinolinate pathways were undetectable, unlike in other neuropsychiatric disorders like ASD [135]. Congruously to these activated production pathways of IAA, increased IAA urinary levels were reported in patients with idiopathic PD [170,171] (Table 6). However, decreased serum IAA were observed in two cohorts of Japanese patients with idiopathic and familial PARK2-mutated PD [172,173], thus showing some inconsistencies across human studies. Some gut bacteria capable of generating IAA are *Klebsiella*, *Ralstonia*, *Staphylococcus*, *Bacillus*, Clostridia, *Bacteroides* and *Escherichia* [135,174] (Table 4). Interestingly, IAA was previously mentioned to suppress pro-inflammatory cytokine production by macrophages and act on the aryl hydrogen receptor (AHR) [109]. IAA was also able to attenuate neuroinflammation in LPS-stimulated BV2 microglia in vitro [175]. Overall, these findings point towards a probable role of altered gut microbial production of IAA in neuroinflammatory processes in PD, even if further investigations need to disentangle the complexities between gut-derived IAA and PD.

The previously mentioned metabolite and uremic toxin p-cresol (or its hepatically sulfonated form p-cresol sulphate) in ASD [142] is generated through intestinal bacterial Tyr metabolization [134], with especially strong producers within Coriobacteriaceae and Clostridium clusters XI and XIVa [137]. P-cresol sulphate has been previously associated with neurological impairments in chronic kidney disease [162]. Moreover, two recent studies reported significantly higher p-cresol sulphate levels in the CSF (yet not in plasma) from PD patients compared to samples from HC [163,176] (Table 6). Additionally, higher CSF to plasma ratios in PD was observed in one study, suggesting that individuals with PD accumulate more p-cresol sulphate in the brain than their healthy counterparts [163,176]. These findings support the relevance of a perturbed BBB allowing the increased permeation of putative neurotoxic microbial metabolites from circulation to the brain in PD [165]. Moreover, p-cresol sulphate levels in CSF associated with the presence of motor fluctuations in PD patients, suggesting a correlative connection with disease progression [163]. This is supported by the fact that p-cresol is a known inhibitor of dopamine–beta-hydroxylase [177], the enzyme facilitating the conversion from DA to norepinephrine. Therefore, alterations in the p-cresol production of the microbiome, as well as the gut bacterial impact on BBB integrity, might regulate neurotransmitter metabolism in the brain. Cirstea et al. [132] have recently provided further evidence that associates altered gut microbial metabolism, disturbed gut function (constipation and IBS) and PD. Compared to HC, PD patients harboured decreased levels of common BA-producing Clostridia, including some Lachnospiraceae genera (*Roseburia*, *Coprococcus)* and *Faecalibacterium*), as well as enriched bacterial clusters associated with p-cresol and phenylacetylglutamine production (*Christensenellaceae*, *Ruminococca*, *Akkermansia*, *Oscillospira*, *Mogibacteriaceae*). Moreover, increased serum levels of p-cresol and phenylacetylglutamine were measured, showing positive correlations with the presence of PD, as well as the severity of gut dysfunction. Therefore, a gut microbiome shift from BA producers to microbes generating AAMs such as p-cresol and phenylacetylglutamine might influence symptoms of intestinal dysfunction, as well as altered circulating metabolites in patients with PD.

Manganese (Mn) has been shown to evoke neurodegenerative processes when accumulated in the brain in the context of PD [178,179]. Recent findings discovered Mn exposure to alter gut bacterial genes involved in amino acid and neurotransmitter metabolism (GABA, glycine, glutamate, Trp, Phe) in a mice model, thus giving rise to the novel proposition of gut bacterial involvement in manganese-associated neurotoxicity [180].

These findings overall suggest that members from the Clostridia class seem to be implicated in the production of AAA metabolites (phenolic and indole derivatives) associated with neurodegenerative disorders. Since Clostridia are also known as key BA producers of the human gut [73], and as the chapters above have discussed, a probable beneficial connection between BA and brain diseases, further investigations in Clostridia-derived metabolites and various brain diseases seem warranted to elucidate the relevance of these bacteria. Furthermore, our collected data on indole and indole derivatives show correlations with various brain disorders (autism, anxiety, PD) that are often accompanied with gut issues [36,110,132,181].

#### 4.1.4. AAMs and Autoimmune Diseases of the Brain

As mentioned above, bacterial production of indole from dietary Trp might be involved in perturbed brain functions. The extensive study by Rothhammer et al. [182] has suggested, that in combination with type I interferons (IFN-Is), gut bacterial-derived metabolites might suppress neuroinflammation through agonistic effects on the aryl hydrogen receptor (AHR) on astrocytes (Figure 3). Serum levels of the AHR agonists indole, indoxyl-(3-)sulphate (IS), IPA and indole-3-aldehyde were found to be lower in patients with MS than in HC (Table 6). Furthermore, experiments conducted in EAE mice models of MS uncovered that depleted dietary Trp exacerbated disease, while the administration of the AHR agonists IS, IPA or indole-3-aldehyde reduced disease burden [182]. IS specifically was further shown to cross the BBB and to stimulate AHR on astrocytes. Not unrelatedly, another bacterial indole derivative, IAA, was able to attenuate neuroinflammation in LPS-stimulated BV2 microglia in vitro [175], which underlines the hypothesis, that bacterial-derived indoles might benefit neuroinflammatory processes by activating the AHR on brain cells. It might further be worth noting that the Trp metabolite and indole derivative IPA was shown to cross the BBB and to ameliorate harmful reactive oxygen species (ROS) in the brain as a neuroprotectant [135]. Described gut genera capable of generating IPA are few and belong to the Firmicutes phyla, namely *Clostridium*, *Peptostreptococcus*, *Escherichia* and *Proteus* [134,135] (Table 4). Therefore, the presence of these IPA-producing genera may indicate beneficial anti-oxidative properties for brain function.

Interestingly, p-cresol producing Clostridiales (including Lachnospiraceae and Ruminococcae families), appeared to be abundant in MS patients’ microbiomes [79,183], which might potentially lead to similar detrimental effects on the CNS as discussed in the chapters on ASD and PD, though no reports on elevated p-cresol in MS patients exist as of now. The only MS study in humans to investigate the faecal metabolome, as far as our literature search was able to capture, provided no relevant findings on non-SCFA bacterial metabolites [29,81]. Future research is encouraged to further develop and confirm these initial findings by conducting metabolomic, gut taxonomical and metagenomic tests in the faecal samples of MS patients in order to look for correlations with bacterial metabolites beyond SCFAs.

### 4.2. Other Metabolites

#### 4.2.1. Trimethylamine N Oxide (TMAO)

The gut bacterial fermentation of dietary L-carnitine and phosphatidylcholine, which are abundant in red meat, produces trimethylamine (TMA). The following hepatic oxidization by flavin-containing monooxygenase 1 and 3 (FMO1 and FMO3) produces trimethylamine N oxide (TMAO), a metabolite frequently linked to increased risk of cardiovascular, metabolic and cerebrovascular disease, whether as a mediating factor, marker or bystander of disease [185,186,187,188]. Recent studies have detected TMAO in human cerebrospinal fluid (CSF), thus establishing its presence in the brain beyond the cerebrovascular system [188,189]. It is to mention that TMAO plasma levels are subject to factors besides gut microbiome composition, namely diet and liver enzyme activity. It is presently unclear how much circulating levels in the CSF depend on the de novo biosynthesis of TMAO in the brain. Recent studies have shown, however, strong correlations between CSF and plasma levels, suggesting that TMAO brain-levels largely derive from the availability in blood, thus supporting the theory of peripheral TMAO reaching the CNS [163]. Interestingly, TMAO plasma levels were found to be significantly higher in elderly humans as well as in aged mice compared to their respective younger groups [190,191,192,193]. This would be coherent with recently reported associations between shifts in gut microbiota and presence of neurodegenerative disorders [89,110,194].

By assessing TMAO CSF levels in volunteers with AD dementia, mild cognitive impairment (MCI) and healthy controls (HC), as well as correlations between TMAO CSF levels and biomarkers of AD and neurodegeneration, Vogt et al. [188] have reported the potential involvement of TMAO in AD. They found significantly higher CSF levels of TMAO in the AD and MCI groups compared to HC, with no differences between AD and MCI [195], all while controlling for age, sex and APOE ε4 genotype. Moreover, they discovered CSF TMAO levels to be significantly correlated with AD biomarkers that indicate a connection to tau pathology and axonal injury. Congruously, a different study observed plasma TMAO levels to be inversely correlated with cognitive functions (working memory, episodic memory and fluid cognition) in middle-aged to older adults [193].

Conversely, a recent study reported no differences in TMAO levels between CSF samples from PD patients and HCs. Nevertheless, significant TMAO elevations were detected in PD patients with motor fluctuations compared to those without (Table 7), thus pointing to a role of TMAO in disease progression [163].

Researchers have previously mentioned shared pathological arteriosclerotic and inflammatory mechanisms between cardiovascular and dementia-associated cerebrovascular diseases [93]. Literature on vascular cognitive impairment (VCI), a broad definition encompassing cognitive disorders with associations to any kind of cerebral vascular brain injury, further proposed that risk factors for VCI, including hypertension, hypercholesterolemia, diabetes mellitus, atrial fibrillation and others, might overlap the ones for AD [196]. Additionally, results in genetically modified mice previously indicated TMAO to cause the progression of atherosclerosis, a risk factor for dementia [195,197]. TMAO was further implicated with decreased reverse cholesterol transport in mice [198] and enhanced platelet hyperreactivity and thrombosis risk in mice and human subjects [199]. This might suggest a vascular aspect of the mechanism by which this bacterial metabolite might take part in the pathophysiology of AD. However, as Vogt et al. [188] found, differences in TMAO levels between healthy controls, MCI and AD groups were independent from traditional cardiovascular disease risk factors such as body mass index, blood pressure, cholesterol and fasting glucose. Positive associations between TMAO levels and biomarkers for AD and neurodegeneration were also further controlled for peripheral vascular disease risks factors, thus implying that TMAO might affect neurodegeneration by other means than vascular mechanisms. Overall, whether and to what extent TMAO might influence AD by the promotion of vascular disfunction is still to be determined at this point in time.

In studies investigating TMAO’s involvement in other mechanisms of neurological diseases, this bacterial metabolite was proposed to weaken the BBB by downregulating tight junction proteins in humans [200]. After reaching the CNS, TMAO was shown to promote neuronal senescence in the hippocampus and cognitive impairment in mice by increasing oxidative stress, disturbing mitochondrial dysfunction and inhibiting the mammalian target of rapamycin (mTOR) signalling, which increased synaptic damage as well as reduced synaptic plasticity-related proteins [190]. A study with the APP/PS1 transgenic mice model of AD indicated higher TMAO-levels in plasma to be associated with cognitive and pathological deterioration, while treatment with the TMA formation inhibitor 3,3-Dimethyl-1-butanol (DMB) alleviated cognitive deterioration and defective synaptic plasticity [191]. Furthermore, DMB treatment managed to reduce hippocampal neuroinflammation and AD-associated pathologies like Aβ42, β-secretase and βCTF (β-secretase-cleaved C-terminal fragment) levels in APP/PS1 mice [191]. Similarly, a different transgenic mouse model of AD (3x Tg-AD) displayed significantly elevated plasma and brain TMAO levels in comparison to healthy controls in a recent study by Govindarajulu et al. [192]. They further incubated hippocampal brain slices of wild-type mice with TMAO and found deficits in synaptic plasticity, impaired synaptic transmission, altered presynaptic and reduced postsynaptic glutamatergic receptor units, as well as induced endoplasmatic reticulum (ER)-mediated protein kinase RNA-like endoplasmic reticulum kinase (PERK) pathway [192]. Another study has further indicated TMAO to impair cognitive function by promoting neuroinflammation and astrocyte activation in mice [193]. In addition to the association between TMAO levels and neuroinflammation and astrocyte activation in older mice, TMAO supplementation in young mice for six months exhibited a decline in memory and learning (assessed through the novel object recognition test) and indeed, elevated markers for neuroinflammation and astrocyte activation. Furthermore, human astrocyte cultures incubated with TMAO showed altered cellular morphology and markers indicating astrocyte activation, thus proposing a direct effect of TMAO on astrocytes [193]. Overall, all of these preclinical findings suggest that TMAO may provoke cognitive impairment by promoting neuroinflammation, AD-related amyloid formation, oxidative stress, as well as deficits in synaptic plasticity and function by promoting ER stress-mediated PERK signalling pathways.

Additionally, an in silico study detected significant correlations between TMAO-related genes and AD biomarkers in nine potential genetic pathways involved in both, that might underline the proposition for TMAO as a strong biomarker for AD [201]. Those nine pathways include in no specific order: the metabolism of proteins; immune system; adaptive immune system; Alzheimer’s disease; axon guidance; amyotrophic lateral sclerosis (ALS); erythropoietin-producing human hepatocellular receptor A (EPHA) and B (EPHB) forward signalling and metabolism of lipids and lipoproteins. These findings might be used as groundwork for investigations of specific pathways that might elucidate a diet-microbial metabolite–brain disease axis.

Curiously, studies in mice and in vitro models reported disease-mediating as well as protective mechanisms by TMAO on processes in neurodegenerative disorders such as a reduction in amyloid aggregation in AD and PD, thus providing a new potential therapeutic target [22,200]. However, more congruous study results have been reviewed and reported on the pro-inflammatory effects of increased TMAO plasma concentrations [200]. These reports underline the need for further studies on TMAO and its combined effects on enhanced circulating pro-inflammatory mediators that can potentially cross a TMAO-induced disturbed BBB to a greater extent, and therefore promote the aforementioned neuroinflammatory and neurodegenerative effects in the brain.

Some gut bacteria found to be significantly associated with enhanced plasma TMAO were the genera *Prevotella*, *Mitsuokella*, *Fusobacterium*, *Desulfovibrio*, *Methanobrevibacter smithii*, and some from the Lachnospiraceae and Ruminococcaceae families [186] (Table 8). Three of those genera belong to the Bacteroidetes and six to the Firmicutes class, congruous with a recent study in dementia [93] that showed a significantly higher Firmicutes/Bacteroidetes ratios in demented individuals with MRI-detected silent lacunar infarctions, as well as strong correlations between dementia and low counts of *Bacteroides* along with higher counts of ‘other bacteria’. Additional sources have reported the following TMA-producing genera: *Anaerococcus*, *Clostridium*, *Escherichia*, *Proteus*, *Providencia* and *Edwardsiella* [22]. Overall, future work is strongly advised to address and investigate TMAO brain levels and any correlations with the gut microbiome.

##### Carnitine Analogues

Given that gut metabolites related to carnitine such as TMAO affect the GBA, researchers have recently discovered two new potentially brain active metabolites, namely the carnitine analogues 3-methyl-4-(trimethylammonio)butanoate (3M4-TMAB) and 4-(trimethylammonio)pentanoate (4-TMAP) [202]. These compounds are generated by gut bacteria from the family Lachnospiraceae (*Clostridiales symbosium* and *clostridioforme*) (Table 8) and were absent in both the gut and brain of GF mice but present in controls, thus suggesting that gut microbiota may be responsible for their presence in the brain. Importantly, these compounds were found in identical regions of white matter of the brain as carnitine and showed an inhibition of carnitine-mediated fatty acid oxidation (FAO) in a murine cell culture model of CNS white matter [202]. FAO is crucial for neuronal energy homeostasis, and inborn errors of this system may link faulty neuronal stem cell self-renewal to ASD [203,204]. Considering these findings and that increased abundance of Clostridia have been linked to ASD and other neurodevelopmental disorders [205], faulty FAO promoted by bacterial metabolites might be an important topic to explore in the future.

#### 4.2.2. Polyphenolic Metabolites

Polyphenols derived from dietary sources are metabolized by human gut microbes to phenolic acids. Research has shown gut microbial metabolites of dietary polyphenols to accumulate in brain tissue and to modulate α-synuclein misfolding, aggregation and neurotoxicity in vitro and in an animal models [206]. Among those metabolites were 3-hydroxybenzoic acid (3-HBA), 3-(3-hydroxyphenyl)propionic acid (3-HPPA) and 3,4-dihydroxybenzoic acid (3,4-diHBA). Further investigations led to the detection of *B. ovatus* as a producer of the aforementioned metabolites that were converted from the dietary polyphenols (+)-catechin (C) and (−)-epicatechin (EC). C/EC-independent production of 3,4-diHBA, 3-HBA and dihydrocaffeic acid (DHCA), a circulating anti-inflammatory phenolic acid, also occurred through *B. ovatus*, *E. lenta* and *E. coli* (Table 8). In earlier studies, 3-HPPA and 3-HBA were shown to ameliorate Aβ-peptide misfolding [207], therefore underlining the potential of bacterial polyphenolic metabolites to protect from neurotoxic protein-aggregation and neurodegenerative disorders like AD and PD. Additional gut bacterial metabolism-derived polyphenols, particularly enerolactone and enterodiol, aryl-γ-valerolactone metabolites and urolithin A/B, exhibit polyphenolic neuroprotective properties [208]. Two studies have further summarized mechanisms by which brain function could be influenced by bioactive microbe-derived metabolites of polyphenols [209,210]. Direct neuroprotective impacts include modulating neuronal receptors, antioxidation, anti-inflammation and overall neuroprotective effects. Indirect mechanisms encompass the modulation of gut microbial homeostasis by supporting beneficial bacteria while decreasing pathogens, as well as improving cerebrovascular health by increased nitrogen oxide levels and vasodilatory response. Limits of the summarized findings were put by their in vitro and ex vivo-based designs.

The ability to cross the BBB is crucial for metabolites to exert neuroactive properties and ten microbe-derived polyphenolic metabolites were found to be capable of distributing in rat brains after intravenous administration [211]: 4-hydroxyhippuric acid, homovanillic acid, 4-hydroxybenzoic acid, vanillic acid, 3-HPPA, trans-ferulic acid, caffeic acid, gallic acid, 3,4-dihydroxyphenyl acetic acid and urolithin B. Taken together, the neuroactive potential of bioactive gut microbe-derived metabolites of dietary polyphenols is promising but the mode of action of these metabolites need further in depth elucidation.

##### Phenolic Compounds

Ferulic acid (FA), a phenolic compound, has been the focus of a handful of studies researching the GBA and has been proposed a role in cognitive development and neuroprotection. Sources of ferulic acid are plants and seeds in the human diet, as well as from gut microbial biosynthesis from dietary cyanidin, catechin and epicatechin. Some beneficial properties of FA are protection from oxidative neurological damage by ROS scavenging, neural stem cell stimulation, and the direct inhibition of Aβ aggregation [8,212,213,214].

Dysfunctions in Pavlovian fear extinction learning are involved in anxiety and fear-associated neuropsychiatric disorders such as PTSD [215,216]. Recently, four bacterial metabolites, of which three were phenolic compounds (phenyl sulphate, pyrocatechol sulphate and 3-(3-sulfooxyphenyl)propanoic acid), have been shown to be significantly decreased in CSF, serum and faecal samples of GF or antibiotic treated mice showing defective fear extinction learning. Further investigations discovered alterations of gene expressions in the medial prefrontal cortex, immature-like microglia, as well as perturbed structural and functional changes in neurons involved in learning processes [168].

#### 4.2.3. Bacterial Amyloid Proteins

Amyloid proteins produced by bacteria have been an emerging subject of interest in the study of pathophysiology in PD [109]. It was reported that bacterial amyloid proteins, such as curli from *E. coli*, could induce the formation of human amyloid aggregates by cross-seeding in a prion-like fashion, as well as promote inflammatory processes by molecular mimicry [217]. The mechanism of cross-seeding was previously shown by curli-induced serum amyloid A amyloidosis in mice [218]. This was further supported by a hypothesis that the phenomenon of protein misfolding in neurodegenerative diseases might originate from the gut, possibly via bidirectional vagal fibres bypassing the circulatory system [108]. It is important to note that vagotomy has been associated with a lower risk for PD [219] and a delayed αSyn dissemination when nervous structures connecting the gut to the brain are severed [220]. Findings of αSyn aggregates in regions beyond the brain, such as the enteric nervous system and olfactory bulb, point to the phenomenon that the majority of PD patients experience gastrointestinal and olfactory dysfunctions long before their diagnosis [221]. Interestingly, hyposmia has been associated with cognitive decline, even in the context of AD [222]. A recent study observed that mice with αSyn overexpression developed PD-like pathological traits after inoculation with curli-producing *E. coli*. These mice displayed αSyn-aggregates in brain and gut tissues, as well as disturbed motor and intestinal functions [223]. Furthermore, prior research reported significantly increased amounts of αSyn aggregates in aged Fischer 344 rat brains and in *Caenorhabditis elegans* after oral inoculation with curli-producing *E. coli* in comparison with subjects colonized with identical strains lacking curli production. Moreover, exposure to curli-producing *E. coli* stimulated immune activity in rat brains, represented by enhanced neuroinflammatory markers, such as upregulated Toll-like receptor 2 (TLR2), interleukin 6, tissue necrosis factor, microgliosis and astrogliosis [224]. The similar immune response pathways to bacterial amyloids by pathogen-associated molecular pattern recognition, and the response to misfolded endogenous amyloids like αSyn and Aβ is intriguing. This illustrates the possibility that gut bacterial amyloids could prime the immune system for a neuroinflammatory response to cerebral amyloid deposits, thus leading to enhanced neuroinflammation and degeneration [217].

Species reported for curli-production are from the family *Enterobacteriaceae*, including *E. coli*, *Salmonella typhimurium*, *Citrobacter spcc.*, *Cronobacter sakazakii* and *Proteus mirabilis.* This was recently deemed as conspicuous by researchers that observed enriched *Enterobacteriaceae spp.* in 31% of PD studies, as well as previous associations between *P. mirabilis* and a PD mouse model [109]. Other residents of the human gut were also reported to generate extracellular amyloids, namely *Streptococcus*, *Staphylococcus*, *Mycobacteria*, *Klebsiella* and *Bacillus spp*. [217]. Overall, these studies provide a hypothesis that bacterial amyloids could be key players in the pathogenesis of neurodegenerative diseases like PD and AD, with data pointing to their effects on amyloid aggregation and on enhanced immunoreactions in the CNS. This subject even motivated Friedland et al. [217] to propose the new term “MAPRANOSIS—the process of microbiota-associated proteopathy and neuroinflammation”. However, some preclinical studies have implicated bacterial amyloids with increased clearance and decreased neuroinflammation by means of activating microglia through the receptor TREM2 [91]. Similarly, toll-like receptors 2 and 4 (TLRs) have shown contradicting effects on amyloid-related neurotoxicity and clearance through microglial activation [225]. It is difficult to draw definite conclusions regarding the effect of gut bacterial amyloids on AD and PD. Nevertheless, current data are strongly pointing to an existing connection.

Yang et al. [226] recently conducted the first study to observe implications of gut dysbiosis and faecal metabolic changes in mice with prions disease, thus providing the base for a new area of research in brain diseases with links to the gut microbiome. Of the previously mentioned metabolites, SCFAs and Trp were decreased, while Tyr increased in mice with prions disease. Furthermore, the microbiome of prion-infected mice harboured distinct compositions from HC, namely enriched Lactobacillaceae, Helicobacteraceae and decreased Prevotellaceae and Ruminococcaceae. Additionally, newly observed altered faecal metabolites consisted of various glycerophospholipids, three secondary bile acids and the toxic avermectin A2b. However, further research should investigate if these compounds are derived from gut bacterial metabolism and whether they are biomarkers or mediators of disease.

## 5. Discussion and Conclusions

Our collected data highlight the fact that research into GBA and the precise role of bacterial metabolites as key contributors is still in its infancy. The majority of studies were conducted in preclinical animal or cell models and only a limited number of human studies are contributing to the current knowledge.

Only a handful of human studies of brain diseases reported faecal SCFAs levels, making it difficult to draw definitive conclusions. Our literature search captured five studies in ASD, two in affective disorders, two in MS, one in PD and none in AD as of this point in time. Nevertheless, an overall decrease in faecal SCFA levels in ASD, affective disorders, MS and PD is apparent (Table 1). Importantly, an increase in one SCFA may be levelled out by the decrease in another SCFA in the same study. AA and PA were reported to be increased in faecal samples [39,42] in two studies, while BA was increased in one [42]. Furthermore, one study observed no significant changes across all SCFAs in ASD [41]. Therefore, it remains to be determined by in-depth studies of the human intestinal metabolome, whether distinct patterns of SCFA levels are present and relevant in different brain diseases.

Based on preclinical findings on how SCFA might impact the brain, the overall assumption points to a beneficial role. The non-exhaustive list of reported effects includes improved gut barrier and BBB integrity [19,20,21,22] and an overall shift towards anti-inflammatory processes [13,28,29,74,75,91]. As HDACI, SCFAs can epigenetically modulate the maturation of brain cells [28,84,87], enhance gene expression for enzymes relevant in catecholamine production [23] and even shift the balance of the immune system towards anti-inflammatory Treg cells and away from pro-inflammatory Th1 and Th17 cells [13,81,83]. Inflammatory processes are known to be involved in MS and to take part in neurodegenerative diseases. As SCFA levels tend to be decreased in most neurodegenerative diseases (Table 1), we might assume that a perturbed gut microbiome might lead to impaired SCFA production of gut bacteria, which might then deplete the beneficial anti-inflammatory effects on the CNS.

Regarding non-SCFA bacterial metabolites, p-cresol has yielded the most human data across studies. It is also the only metabolite with measurements in faecal samples that correlated with brain disease, namely in patients with ASD. Taken all results together (Table 6), p-cresol is significantly increased in faecal, urinary and blood samples of ASD, as well in the CSF and serum of PD patients. CSF and plasma levels of p-cresol have moreover shown to be correlated with the severity of PD [163]. This, and preclinical studies in mice models implicating p-cresol with detrimental effects on the CNS [140,141], seem to be in line with elevated levels in ASD and PD. The notion of involvements of p-cresol in ASD is supported by the finding that 4EPS, a metabolite with structural similarities to p-cresol, induces ASD-behaviour in mice.

Alterations in Trp metabolism were observed in human studies in ASD, PD and MS. Significant assessments of Trp metabolites such as indole and indole derivatives are mainly from non-faecal samples and have shown an overall increase in ASD, a decrease in MS and inconsistent results in PD (Table 6). Nevertheless, it is important to mention that two PD studies showed indole and indole derivatives to be increased in CSF [109,163], a compartment closely connected to brain tissue. Furthermore, metagenomic tests concluded that overall Trp metabolism pathways are enriched in the gut microbiome of ASD patient cohorts, and that indole pathways are enhanced in PD microbiomes [135]. These metagenomic findings and the several reports on metabolite levels in patients seem to indicate the presence of perturbed and enriched Trp metabolism in ASD and PD. Naturally, the lack of studies testing for faecal metabolites in humans, as well as the inconsistencies within results in PD stress the limitation to unequivocally determine distinct patterns by which our gut bacteria alter Trp metabolism in various brain diseases.

Several probable mechanisms by which Trp metabolites might exert their impact were identified. Preclinical studies have indicated that indole and its derivatives (indole, IS, IPA, indole-3-aldehyde, IAA) might actually be able to limit neuroinflammation by acting as agonists on the AHR [109,182] (Figure 3). Furthermore, a shift in Trp-metabolism away from 5-HT production might partly explain the worsening of disease in individuals who are responsive to treatment with selective serotonin-reuptake inhibitors (SSRIs) [146,147]. Thus, a decrease in Trp availability might be connected to brain diseases, which is further exemplified by Trp-depletion experiments in depressed human cohorts and mice models of MS [146,147,182]. It might further be of interest that IPA was reported to ameliorate toxic ROS activity in the brain [135]. On the other hand, IS has induced oxidative stress in animal and in vitro studies [163,164], as well as displayed potential neurotoxic effects in mice through perturbed microglial and astrocyte function [165]. Moreover, an overproduction of indole was associated with anxiety and depression levels in rats [155]. Taken together, these results imply that alterations in the gut bacterial metabolism of AAA, whether it is Tyr-derived p-cresol or Trp-derived indole metabolites, could contribute to brain diseases.

The question as to whether gut bacterial amyloids like curli might contribute to or alleviate diseases with an accumulation and aggregation of misfolded proteins and neuroinflammation, cannot be unequivocally answered yet. Nevertheless, the current evidence on bacterial amyloids implies that there might be more to bacterial metabolites with connections to the brain beyond SCFAs and AAM.

The majority of evidence presented here is derived from preclinical studies, such as in vivo studies in transgenic animals, in germ-free animals or animals exposed to early-life alterations of the gut microbiota including pathogens, probiotics, or antibiotics. The validity of drawing decisive conclusions for the human physiology from animal studies is therefore conceivably limited. Moreover, the information on taxonomical alterations and associations to metabolites and diseases is non-exhaustive since this study focused on bacterial metabolites. The objective was to assess bacterial metabolites independently as probable determinants of disease, thus shifting the focus away from their producers. The need for further work in deciphering the vast and intricate correlations between gut microbial communities, faecal metabolites and the presence of brain dysfunction, is definitely acknowledged and deemed as necessary.

Results regarding the potential protective or aggravating role of bacterial metabolite groups on brain diseases are still few and require additional confirmation. A final assessment of the importance of faecal metabolomic changes in brain diseases, however, is problematic as of now. The difficulties lie in the heterogeneity of disease manifestations and varying technological methods to assess varying sample sources. Furthermore, confounders like gender, genetics, dietary factors, medication and lifestyle, as well as subtypes of bacterial species, might contribute to insignificant or falsified results. Therefore, additional independent research applying methodical standardization is essential to ensure comparable and reproducible data. Determining not only taxonomical data, but also conducting functional analyses through metagenomic and metabolomic testing of faecal samples would crucially increase the robustness of discovered associations. This might further develop, confirm or refute today’s initial findings on correlations between bacterial metabolites and brain diseases. Interests lie in the discovery and unravelment of GBA mechanisms, as well as the study of bacterial metabolites as promising key contributors to brain diseases. Successful findings of such might be crucial in identifying aetiological and pathophysiological processes, thus efficaciously supporting future research in novel treatments and the prevention strategies of brain diseases.

## Figures and Tables

**Table 1:** SCFA level alterations in brain diseases found in human studies.

Disease	SCFA		Literature	*p*-Values
ASD	AA	↓	f [44], f [43]	***p* = 0.011, *p* = 0.0000003**
		↑	f [42], f [39],* u [36]	***p* = 0.037, *p* < 0.005, *p* < 0.005**
		-	f [41]	*p* = 0.979
	BA	↓	f [44], f [43]	***p* = 0.005, *p* = 0.005**
		↑	f [42]	***p* = 0.025**
		-	f [41]	*p* = 0.974
	Isobutyric acid	↑	f [42]	***p* = 0.022**
	Isovaleric acid	↑	f [42]	***p* = 0.038**
	PA	↓	f [43]	***p* = 0.002**
		↑	f [42], f [39]	***p* = 0.007, *p* < 0.005**
		-	f [41], f [44]	*p* = 0.979, *p* = 0.243
	VA	↓	f [43]	***p* = 0.005**
		↑	f [44], f [42]	***p* < 0.001, *p* = 0.007**
MDD	AA	↓	f [67]	***p* = 0.04**
		↑	f [69]	*p* = 0.65
	BA	-	f [67], f [69]	*p* = 0.68, *p* = 0.867
	Caproic acid	↑	f [67]	*p* = 0.09
	Isobutyric acid	-	f [67]	*p* = 0.70
		-	f [69]	*p* = 0.501
	Isocaproic acid	↑	f [67]	***p* < 0.01**
	Isovaleric acid	-	f [67]	*p* = 0.4
	PA	↓	f [67]	***p* = 0.07**
		-	f [69]	*p* = 0.918
	VA	↓	f [67]	*p* = 0.56
MS	AA	↓	f [81], s [29]	***p* < 0.0001, *p* = 0.001**
	BA	↓	f [81], s [29]	***p* < 0.05, *p* = 0.0001**
	Isovalerate, valerate, hexanoate, heptanoate	-	s [29]	*p* > 0.05
	PA	↓	f [81], s [29]	***p* < 0.0001, *p* = 0.01**
PD	AA	↓	f [117], * p [109]	***p* < 0.01, *p* = 0.0201**
	BA	↓	f [117]	***p* < 0.01**
	Isobutyric acid	-	f [117]	*p* > 0.05
	Isovaleric acid	-	f [117]	*p* > 0.05
	PA	↓	f [117]	***p* < 0.01**
	VA	-	f [117]	*p* > 0.05

This table shows the differences of SCFA levels of various sample materials from human patients compared to healthy controls. Significance of the data is given in the last column. ↑ symbolizes increased, ↓ decreased, whereas - symbolizes no significant change in metabolite levels found in cohorts with the specific disease. Sample material is noted as f *=* faecal; s *=* serum; p *=* plasma; u *=* urine with the associated reference as numbers in brackets; *p*-values < 0.05 are marked in bold letters. BA *=* butyric acid; AA *=* acetic acid; PP *=* propionic acid; VA *=* valeric acid; ASD = autism spectrum disorder; MDD = major depressive disorder; MS = multiple sclerosis; PD = Parkinson’s disease. “*” marked references are sourced from reviews.

**Table 2:** Gut-residing bacteria found to correlate with the production of SCFAs.

SCFA	Taxa	Study
**tSCFA**	*Faecalibacterium*, *Ruminococcus*, *Bifidobacterium*	[39]
**PA**	*Bacteroides*	[39]
**VA**	Acidobacteria, Actinomycetaceae	[44]
**BA**	Streptococcaceae, Peptostreptococcaceae, Lactobacillaceae, Clostridiaceae, Family_XIII, Leuconostocaceae	[44]
**PA**	Desulfovibrionaceae, Streptococcaceae	[44]
**AA**	Desulfovibrionaceae	[44]
**AA, PA**	*Prevotella_9*	[81]
**BA**	*Clostridium*, *Eubacterium*, *Butyrivibrio*	[103]
**AA**	*Bacteroidetes*, *B.hydrogenotrophica*	[130]
**BA**	Lachnospiraceae, *Faecalibacterium prausnitzii*, *Eubacterium*, *Roseburia*	[130]
**PA**	*Bacteroidetes*, Proteobacteria, some Lachnospiraceae	[130]
**BA**	*Eubacterium ramulus*	[131]
**PA**	*Clostridium*	[46]
**AA, PA**	*Parabacteroides distasonis*, *Megaspheara massiliensis*	[87]
**BA, VA, HA**	*Parabacteroides distasonis*, *Megaspheara massiliensis*	[87]
**PA**	*Lactobacillus*, *Propionibacterium*	[97]
**BA**	*Faecalibacterium prausnitzii*, *Eubacterium rectale*, *Roserburia*, *Eubacterium hallii*, *Ruminococcus bromii*	[14]
**PA**	*Akkermansia municiphila*	[14]
**BA**	*Blautia*, Lachnospiraceae: *Coprococcus*, *Roseburia*, *Faecalibacterium*, *Lachnospira*	[132]
**BA**	Clostridia (class)	[73]
**AA**	*Blautia hydrogenotrophica*, *Clostridium*, *Streptococcus*	[82]
**PA**	*Salmonella*, *Roseburia inulinivorans*, *Ruminococcus obeum*, *Bacteroides*, *Phascolarctobacterium succinatutens*, *Dialister*, *Veillonella*, *Megasphaera elsdenii*, *Coprococcus catus*	[82]
**BA**	*Anaerostipes*, *Coprococcus catus*, *Eubacterium rectale*, *Eubacterium hallii*, *Faecalibacterium prausnitzii*, *Roseburia*, *Coprococcus comes*, *Coprococcus eutactus*	[82]

tSCFA *=* total SCFAs; BA *=* butyric acid; AA *=* acetic acid; PP *=* propionic acid; VA *=* valeric acid; HA *=* hexanoic acid. References are represented by numbers in brackets.

**Table 3:** Prevalence of dementia linked with various factors.

Factors	Odds Ratio	*p*-Value
Enterotype III	18.5 ^b^	<0.001 ^b^
Enterotype I	0.1 ^a^	<0.001 ^a^
ApoE	3.9 ^a^, 4.4 ^b^	0.035 ^a^, 0.026 ^b^
SLI	15.0 ^a^	0.005 ^a^
VSRAD	3.5 ^a^, 4.2 ^b^	<0.001 ^a,b^

Multivariable logistic regression analysis models linking the prevalence of dementia and various factors from Saji et al. [93]. ^a^ Model 1: inclusion of enterotype I, ^b^ Model 2: Inclusion of enterotype III. Abbreviations: ApoE ε4 = apolipoprotein ε4; SLI = silent lacunar infarct; VSRAD = voxel-based specific regional analysis system for Alzheimer’s disease.

**Table 4:** Gut-residing bacteria found to correlate with the production of amino acid metabolites (AAMs).

AAM	Taxa	Study
Taurine	*Alistipes HGB5*, *Alistipes finegoldii*, *Bacteroides xylanisolvens*	[45]
GABA	*Bifidobacterium*, *Bacteroides*, *Lactobacillus*; *Lactobacillus brevis*	[41,97]
GABA, lactate	*E. coli HT115-strain*	[144]
Serotonin	*Candida*, *Streptococcus*, *Escherichia*, *Enterococcus*, *Pseudomonas*	[135,184]
Serotonin, dopamine, norepinephrine	*Streptococcus*, *Enterococcus*, *Escherichia*	[135]
Serotonin, dopamine	*Clostridiales incertae sedis*	[150]
Norepinephrine	*Escherichia*, *Bacillus*, *Saccharomyces*	[184]
Dopamine	*Bacillus*	[184]
Acetylcholine	*Lactobacillus*	[184]
4EPS, p-cresol (sulphate)	*Clostridium*	[36,140,142]
P-cresol (sulphate)	Clostridiaceae (*Clostridium I*, *IV*, *IX*, *XI*, *XIII*, *XIVa*, *XVI*), Bacteroidaceae, Coriobacteriaceae	[109,137]
P-cresol, phenylacetylglutamine	*Oscillospira*, *Ruminococcus*, Mogibacteriaceae, Christensellaceae, Clostridiales, *Akkermansia*	[132]
Dextrorphan O-glucuronide, 3-methyldioxyindole(F4)	*Christensenella*, *Candidatus arthromitus*	[150]
“**TRYP6**” (Kynurenine, quinolinate, indole, IAA, IPA, tryptamine)	Actinobacteria, Firmicutes, Proteobacteria, Bacteroidetes, Fusobacteria	[135]
Quinolinate, indole, IAA, IPA, tryptamine	*Clostridium*	[135]
Kynurenine, quinolinate, indole, IAA, IPA	*Burkholderia*	[135]
Kynurenine, quinolinate, IAA, tryptamine	*Streptomyces*, *Pseudomonas*, *Bacillus*	[135]
IAA	*Bacillus*, *Klebsiella*, *Ralstonia*, *Staphylococcus*	[135]
Indole	*Bacteroides*, *Citrobacter*, *Clostridium_XIX*, *Desulfitobacterium*, *Edwardsiella*, *Escherichia*, *Fusobacterium*, *Providencia*, *Shigella*	[135]
	*Parabacteroides distasonis*, *Megasphaera massiliensis* *E. coli*	[87] [155]
IPA	*Clostridium*, *Escherichia*, *Proteus*	[135]
Kynurenine	*Pseudomonas*, *Bacillus*, *Burkholderia*, *Streptomyces*	[135]
Quinolinate	*Klebsiella*, *Bacillus*, *Burkholderia*	[135]
Tryptamine	*Holdemania*, *Tyzzerella*, *Desulfovibrio*, *Yersinia*, *Bacillus*, *Clostridium*, *Ruminococcus*	[135]
Indole, indoxyl-(3)-sulphate, IPA, indole-(3)-aldehyde	*Lactobacillus reuteri*	[182]

GABA = gamma-aminobutyric acid; TRYP6 = six Trp metabolism pathways generating the neuroactive metabolites in brackets (“); 4EPS = 4-ethylphenylsulfate; IAA = indole-acetic acid; IPA = indole-propionic acid. References are listed as numbers in brackets.

**Table 5:** Sixteen faecal metabolites with significant correlation in rats with induced depression (adjusted from Yu et al. [150]).

Faecal Metabolites	Trend in Depressed Rats
Nicotinic acid	↑
Hypoxanthine	↑
Dextrorphan O-glucuronide	↑
3-Methoxytryptophan	↑
5-Methoxytryptophan	↓
L-Urobilin	↑
MG(0:0/20:3(5Z,8Z,11Z)/0:0)	↓
PE(14:1(9Z)/14:1(9Z))	↑
PS(18:0/22:6(4Z,7Z,10Z,13Z,16Z,19Z))	↑
Cholic acid	↑
MG(0:0/20:4(8Z,11Z,14Z,17Z)/0:0)	↓
Stearyl citrate	↓
Hyocholic acid	↑
L-Urobilinogen	↑
Deoxycholic acid	↑
Chenodeoxycholic acid	↑

Faecal levels in depressed rats compared to healthy control group: ↑ = upregulated; ↓ = downregulated. Rows marked in blue have a *p*-value of *p* < 0.01, whereas rows in white reached *p* < 0.05. MG = monoacylglyceride; PE = phosphatidylethanolamine; PS = phosphatidylserine

**Table 6:** Bacterial AAMs correlated with brain diseases or the progression of brain diseases.

Disease	Amino Acid Metabolites		Sample and Literature	*p*-Values
ASD	P-cresol	-	f [42]	*p* = 0.884
		↑	f [39], f [41], * s/p [36], u [138,139]	***p* < 0.05, *p* = 0.04, *p* < 0.05, *p* < 0.05, *p* < 0.05**
	IAA, indolyl lactate	↑	u [143]	***p* < 0.001**
	IS	↑		***p* < 0.05**
	Indoles (indole, 3-methylindole)	↑	f [39]	***p* < 0.05**
	Serotonin, GABA	↑	* s/p [36]	***p* < 0.05**
	GABA	↓	f [41]	*p* = 0.077
	3-(3-hydroxyphenyl)-3-hydroxypropionic acid, 3-hydroxyphenylacetic acid, 3-hydroxyhippuric acid	↑	* u [36]	***p* < 0.05**
PD	P-cresol	↑	* c [109], c [176], s [132], cc^a^p^a^ [163]	***p* < 0.05, *p* = 0.0002, *p* = 0.0028, *p* < 0.05**
	IAA	↓	s [172,173], * s [109]	***p* = 0.0083, *p* = 0.0258, *p* < 0.05**
		↑	* cp [109], u [170,171]	***p* < 0.05, *p*^b^, *p* < 0.001**
	Indole	↑	* cp [109]	***p* < 0.05**
	IS	↑	c ^a^ [163]	***p* < 0.05**
	Catechol sulphate, hippuric acid, 3-hydroxyhippuric acid, catechol sulphate, 3-(3-hydroxyphenyl)propionic acid, indole-3-methyl acetate, 2-furoylglycine, phenylethylamine	↓	* s [109]	***p* < 0.05**
	Phenylactate, 3-(4-hydroxyphenyl)lactate	↑	* s [109]	***p* < 0.05**
	3-(4-hydroxyphenyl)acetic acid, tryptamine, phenylacetic acid, aminobenzoic acid, hydroxybenzoic acid	↑	u [170,171]	***p* < 0.05**
	Phenylacetylglutamine	↑	s [132]	***p* = 0.004**
	Quinic acid	↑	* c [109]	***p* < 0.05**
	Trimethylamine, threonate	↓	* p [109]	***p* < 0.05**
	Benzoic acid, 3-(4-hydroxyphenyl)acetic acid	↓	* cp [109]	***p* < 0.05**
MS	Aryl hydrocarbon receptor agonists	↓	s [182]	***p* < 0.05**

This table shows the differences of AAM levels of various sample materials from human patients compared to healthy controls. *p*-values are listed in the last column while the significant date is written in bold letters. ↑ symbolizes increased, ↓ decreased, whereas—symbolizes no change in the metabolite levels found in cohorts with the specific disease. Sample material is noted as f *=* faecal; s *=* serum; c *=* cerebrospinal fluid; p *=* plasma; u *=* urine; s/p *=* blood samples with the associated reference as numbers in brackets. References noted with “*” are sourced from reviews. *p*-values < 0.05 are marked in bold letters. ^a^ correlated with progression of disease. ^b^ (*p* = 0.00364 PD in early stage of disease, *p* < 0.001 PD in mid-stage, *p* = 0.056 PD in late-stage compared to HC). (For example: cc^a^p^a^ = altered cerebrospinal fluid sample, and altered cerebrospinal as well as plasma samples which correlate with disease progression). IAA *=* indoleacetic acid; IS *=* indoxyl sulphate; GABA *=* gamma-aminobutyric acid; IPA *=* indole propionic acid.

**Table 7:** Other bacterial metabolites correlated with brain diseases or progression in humans.

Disease	Metabolite	Change	Literature
AD	TMAO	↑	c [188]
MCI	TMAO	↑	c [188]
PD	TMAO	↑	c^a^pp^a^ [163]
ASD	Isopropanol	↑	f [41]

AD = Alzheimer’s disease; MCI = mild cognitive impairment; PD = Parkinson’s disease, ASD = autism spectrum disorder; TMAO = trimethylamine N oxide, ^a^ correlated with the progression of disease. Significance of data is *p* ≤ 0.05. ↑ symbolizes increased metabolite levels found in cohorts with the specific disease compared to healthy controls. Sample material is noted as f = faecal, c = cerebrospinal fluid, p = plasma sample with the associated reference as numbers in brackets.

**Table 8:** Gut-residing bacteria found to correlate with the production of other metabolites.

Other Metabolites	Taxa	Study
TMA(O)	*Prevotella*, *Mitsuokella*, *Fusobacterium*, *Desulfovibrio*, *Methanobrevibacter smithii*; some from Lachnospiraceae and Ruminococcaceae	[186]
TMA	*Anaerococcus*, *Clostridium*, *Escherichia*, *Proteus*, *Providencia*, *Edwardsiella*	[22]
curli	Enterobacteriaceae (*E. coli*, *Salmonella typhimurium*, *Citrobacter freundii*, *Cronobacter sakazakii*, *Proteus mirabilis*)	[109]
curli	*E. coli*	[224]
curli	*Streptococcus*, *Staphylococcus*, *Mycobacteria*, *Klebsiella*, *Bacillus*	[217]
Nicotinamide	*Akkermansia muciniphila*	[227]
3-HBA, 3,4diHBA, DHCA	*Bacteroides ovatus*	[206]
3-methyl-4-(trimethylammonio)butanoate, 4-(trimethylammonio)pentanoate	Lachnospiraceae (Clostridiales): *C.clostridioforme*, *C.symbosium*	[202]

TMA(O) *=* trimethylamine N oxide; TMA *=* trimethylamine; 3-HBA *=* 3-hydroxybenzoic acid; 3,4-diHBA *=* 3,4-dihydroxybenzoic acid; DHCA *=* dihydrocaffeic acid. References are represented by numbers in brackets.

## Data Availability

Data sharing not applicable. No new data were created or analyzed in this study. Data sharing is not applicable to this article.

## Abbreviations

CNS	central nervous system
SCFA	short-chain fatty acids
AAM	amino acid metabolites
ASD	autism spectrum disorder
MS	multiple sclerosis
PD	Parkinson’s disease
AD	Alzheimer’s disease
GIT	gastrointestinal tract
GBA	gut–brain axis
BBB	blood–brain barrier
BA	butyric acid
PA	propionic acid
AA	acetic acid
VA	valeric acid
TMAO	trimethylamine N-oxide
IS	indoxyl sulphate
4EPS	4-ethylphenylsulfate
IAA	indole acetic acid
IPA	indole propionic acid
IBS	irritable bowel syndrome
GF	germ free
WT	wild type
HDACI	histone deacetylase inhibition/inhibitor
NT	neurotypical
FMT	faecal microbiome transplants
HC	healthy controls
DBH	dopamine beta-hydroxylase
MDD	major depressive disorder
HAB	high anxiety-like behaviour
NaB	sodium butyrate
αSyn	alpha-synuclein protein
AAA	aromatic amino acids
Tyr	tyrosine
Phe	phenylalanine
Trp	tryptophan
5-HT	serotonin
ATD	acute tryptophan depletion
DA	dopamine
AHR	aryl hydrogen receptor
